# Heterotrophic Sulfur Oxidation of *Halomonas titanicae* SOB56 and Its Habitat Adaptation to the Hydrothermal Environment

**DOI:** 10.3389/fmicb.2022.888833

**Published:** 2022-06-14

**Authors:** Rui Du, Di Gao, Yiting Wang, Lijun Liu, Jingguang Cheng, Jiwen Liu, Xiao-Hua Zhang, Min Yu

**Affiliations:** ^1^College of Marine Life Sciences, and Frontiers Science Center for Deep Ocean Multispheres and Earth System, Ocean University of China, Qingdao, China; ^2^Laboratory for Marine Ecology and Environmental Science, Pilot National Laboratory for Marine Science and Technology, Qingdao, China; ^3^Institute of Evolution and Marine Biodiversity, Ocean University of China, Qingdao, China

**Keywords:** *Halomonas titanicae*, comparative genomic, metabolic analysis, sulfur-oxidizing bacteria, environmental adaptation

## Abstract

*Halomonas* bacteria are ubiquitous in global marine environments, however, their sulfur-oxidizing abilities and survival adaptations in hydrothermal environments are not well understood. In this study, we characterized the sulfur oxidation ability and metabolic mechanisms of *Halomonas titanicae* SOB56, which was isolated from the sediment of the Tangyin hydrothermal field in the Southern Okinawa Trough. Physiological characterizations showed that it is a heterotrophic sulfur-oxidizing bacterium that can oxidize thiosulfate to tetrathionate, with the Na_2_S_2_O_3_ degradation reaching 94.86%. Two potential thiosulfate dehydrogenase-related genes, *tsdA* and *tsdB*, were identified as encoding key catalytic enzymes, and their expression levels in strain SOB56 were significantly upregulated. Nine of fifteen examined *Halomonas* genomes possess TsdA- and TsdB-homologous proteins, whose amino acid sequences have two typical Cys-X2-Cys-His heme-binding regions. Moreover, the thiosulfate oxidation process in *H. titanicae* SOB56 might be regulated by quorum sensing, and autoinducer-2 synthesis protein LuxS was identified in its genome. Regarding the mechanisms underlying adaptation to hydrothermal environment, strain SOB56 was capable of forming biofilms and producing EPS. In addition, genes related to complete flagellum assembly system, various signal transduction histidine kinases, heavy metal transporters, anaerobic respiration, and variable osmotic stress regulation were also identified. Our results shed light on the potential functions of heterotrophic *Halomonas* bacteria in hydrothermal sulfur cycle and revealed possible adaptations for living at deep-sea hydrothermal fields by *H. titanicae* SOB56.

## Introduction

Deep-sea hydrothermal vents are characterized by darkness, high pressures, and steep gradients of chemical factors, such as high concentrations of reduced compounds, gases (H_2_, CH_4_, and H_2_S), and heavy metals (copper, cadmium, and lead) (Patwardhan et al., 2018; Dick, 2019), in the mixing regions between hot hydrothermal fluid and cold deep-sea water. These thermal and chemical gradients provide a wide range of niches for microbial communities living in and around vents (Nakagawa and Takai, 2008; Kentaro and Ken, 2014; Dick, 2019). Inorganic sulfur compounds, including hydrogen sulfide, elemental sulfur, thiosulfate, and polysulfide, are enriched both in the mixing zone and far away from vents (Mullaugh et al., 2008; Gartman et al., 2011; Beinart et al., 2015). Following the discovery of deep-sea hydrothermal ecosystems in 1977, the microbial populations capable of ammonia oxidation, nitrite oxidation, sulfur oxidation, and manganese oxidation were detected to be the main chemolithoautotrophs to sustain primary production in these unique ecosystems (Fortunato et al., 2018; Dick, 2019; Yang et al., 2019). In addition, partially oxidized inorganic sulfur compounds serve as both electron donors and acceptors in a variety of energy metabolism processes (Yamamoto and Takai, 2011). Therefore, the chemical and microbial oxidation and reduction of sulfur compounds are probably responsible for the establishment of the complex sulfur-metabolic network of hydrothermal ecosystems.

Sulfur oxidation is prevalent in hydrothermal systems, as sulfur is often a dominant electron donor in hydrothermal vents at fast-spreading ridges, and also key electron sources for microbial metabolism (Dick, 2019). The element sulfur can exist in various valence states ranging from −2 to +6, which results in a variety of reduced inorganic sulfur compounds including tetrathionate (S_4_O_6_^2−^), thiosulfate (S_2_O_3_^2−^), sulfite (SO_3_^2−^), sulfide (S^2−^), and elemental sulfur (S^0^). A variety of enzymes and proteins involved in the oxidation of sulfur compounds were discovered, including sulfur-oxidizing enzymes, sulfur transferases, and sulfur carrier proteins (Wang et al., 2018a). As one of thiosulfate oxidation pathways, the S_4_ intermediate (S_4_I) pathway is made up of a thiosulfate:quinol oxidoreductase (Tqo or DoxDA) and a tetrathionate hydrolase (TetH). DoxDA oxidizes thiosulfate to tetrathionate, while TetH hydrolyzes tetrathionate to thiosulfate and other products (Ghosh and Dam, 2009; Wang et al., 2018a). S_4_I pathway is widely found in many chemoautotrophic genera including *Acidithiobacillus*, *Thermithiobacillus*, *Halothiobacillus*, and *Tetrathiobacter* (Dam et al., 2007; Ghosh and Dam, 2009; Wang et al., 2018a), however, few are found in heterotrophic bacteria. It was reported that thiosulfate dehydrogenases (TsdA) also catalyze the oxidation from thiosulfate to tetrathionate (Mandal et al., 2020). In this reaction, thiosulfate is oxidized in the periplasm by thiosulfate dehydrogenase, which requires *c*-type cytochrome as an electron acceptor and/or a c-type heme molecule in the protein (Denkmann et al., 2012; Kurth et al., 2016; Jenner et al., 2019). Several thiosulfate-oxidizing and tetrathionate-forming thiosulfate dehydrogenases have been identified and characterized in both chemolithotrophic and phototrophic sulfur bacteria. Notably, the capability of oxidizing thiosulfate to tetrathionate is widely observed among chemo- and organo-heterotrophic bacteria, haloarchaea (Sorokin et al., 2005) and yeasts. For example, the genera *Pseudomonas*, *Halomonas*, and *Bacillus* were all reported to possess the tetrathionate-forming ability (Sorokin, 2003; Mandal et al., 2020). By forming tetrathionate, these microorganisms may use thiosulfate as a supplemental inorganic energy source and may actively participate in sulfur cycling when thiosulfate is present.

*Halomonas*, the largest genus of the family *Halomonadaceae*, are comprised of 143 species with validly published names (http://www.bacterio.net/index.html), and most of them were found in saline environments, including marine (Xu et al., 2013), salterns (Zhao et al., 2012), saline lakes and soils (Poli et al., 2013), and in salty foods (Arahal and Ventosa, 2006). Bacteria from the genus *Halomonas* showed optimal growth under conditions of 0.5–2.5 M NaCl and exhibited an extraordinary ability to rapidly adapt to changes in the external salt concentration (Mata et al., 2002). Within *Halomonas*, the accumulation of compatible solutes under high osmotic pressure conditions is a universal adaptation mechanism, and these substances mainly include glycine, betaine, ectoine, and hydroxyectoine (Grammann et al., 2002; Kushwaha et al., 2019). Over the last decade, interests in *Halomonas* species have been centered on their ability to produce exoenzymes, exopolysaccharides, and other commercially valuable products (Bouchotroch et al., 2000; Mata et al., 2006; Chen et al., 2017), whereas few studies have focused on their sulfur-oxidizing activities and ecological functions in extreme environments.

The Okinawa Trough is a back-arc basin located adjacent to the west of the Ryukyu trench-arc system, along the eastern margin of Eurasian continent (Miyazaki et al., 2017). Several hydrothermal fields have been discovered in the middle and southern parts of the Okinawa Trough. The hydrothermal fluids in the Okinawa Trough are characterized by high alkalinity and abundant carbon dioxide, hydrogen sulfide, and methane, and sediments are characteristically polymetallic (Miyazaki et al., 2017). In hydrothermal environments, sulfur oxidation is the major energy source for primary production, which is mainly conducted by chemolithoautotrophic microorganisms that are either free-living or associated with invertebrates as symbionts (Sievert et al., 2008). It has been reported that sulfur-oxidizing bacteria show a relatively high abundance in hydrothermal deposits of the Okinawa Trough (Wang et al., 2018b). Although autotrophic sulfur-oxidizing bacteria play an important role in biogeochemical cycle of hydrothermal vents, sulfur metabolism conducted by heterotrophic bacteria is also ubiquitous in these environments. The adaptation mechanism of heterotrophic sulfur-oxidizing bacteria and their survival strategies in deep-sea hydrothermal environments need to be further revealed.

In this study, the sulfur-oxidizing bacterium *H. titanicae* SOB56, which can grow aerobically in artificial seawater (ASW) medium, was isolated from sediment of the Tangyin hydrothermal field in the Okinawa Trough. General genomic features were elucidated through comparative genomic analysis, and its sulfur-oxidizing ability and essential genes involved were identified in a series of experiments and bioinformatic analyses. Combined with the results of comparative genomic analysis and laboratory experiments, its capabilities in sulfur oxidation and adaptation to micro-niches in the hydrothermal environment were summarized.

## Materials and Methods

### Bacterial Growth and DNA Extraction

Strain *H. titanicae* SOB56 was isolated from the sediment of the Tangyin hydrothermal field in the Okinawa Trough using a standard dilution plating technique (Li et al., 2019) on the modified ASW medium. The composition of ASW medium for sulfur-oxidizing bacterial growth was as follows (L^−1^): 23.0 g NaCl, 11.6 g MgCl_2_•6H_2_O, 4.0 g Na_2_SO_4_, 1.1 g CaCl_2_, 0.6 g KCl, 0.2 g NaHCO_3_, 1.0 g NH_4_Cl, 0.05% potassium phosphate solution (pH 7.3), 0.1 mg vitamin B_12_, and 1 ml trace element solution. The trace element solution contained the following (L^−1^): EDTA 50 g, ZnSO_4_•7H_2_O 22 g, CaCl_2_ 5.54 g, MnCl_2_•4H_2_O 5.06 g, FeSO_4_•7H_2_O 4.99 g, (NH_4_)_6_•Mo_7_•4H_2_O 1.10 g, CuSO_4_•5H_2_O 1.57 g, and CoCl_2_•6H_2_O 1.61 g. The pH of trace element solution was adjusted to 6.0 with KOH (Ruby et al., 1981). ASW was finally supplemented with 10 mM sterile filtered Na_2_S_2_O_3_ solution as sulfur source and phenol red as a pH indicator. Incubation was performed in the dark at 28°C, and the color change of medium was used to determine the metabolic type of *H. titanicae* SOB56. The genomic DNA was extracted by using the phenol-chloroform-isoamylic alcohol extraction protocol described by Yin et al. (Yin et al., 2013). The 16S rRNA gene was amplified by PCR using bacterial primers 8F (5′-AGAGTTTGATCCTGGCTCAG-3′) and 1492R (5′-GGTTACCTTGTTACGACTT-3′) (Yin et al., 2013). The 30 μl PCR reactions included 3 μl dNTP (2 mM), 0.3 μl of each primer (20 μM), 0.05 U Taq polymerase (Fermentas International Inc), and 3 μl 10× Taq buffer. The PCR was performed for 5 min at 95°C, followed by 30 cycles of 1 min at 95°C, 60 s at 55°C, and 90 s at 72°C. A final extension step of 10 min at 72°C was used. The PCR products of the 16S rRNA gene and genomic DNA were both sent to Beijing Genomics Institute (BGI, Shenzhen, China) for sequencing.

### Genome Sequencing, Analysis, and Annotation

Genomic DNA of *H. titanicae* SOB56 was sequenced on the Illumina HiSeq 4,000 platform with a 270 bp paired-end library and the PacBio RSII platform. 718 Mb and 610 Mb clean reads were obtained from Illumina and PacBio platforms after filtering with SOAP nuke (Chen et al., 2018) and SMRT Analysis version 2.2.0 (https://smrt-analysis.readthedocs.io/en/latest/SMRT-Analysis-Release-Notes-v2.2.0/) using default parameters, respectively. To obtain better *de novo* assembly results, the two datasets of clean reads were combined for a hybrid assembly. A total of 5,279,693 bp complete genome was obtained after assembling and polishing using Canu v1.1 (Koren et al., 2017) and Pilon v1.16 (Walker et al., 2014) with default parameters. Genes were initially predicted and annotated by RAST Server pipeline (Aziz et al., 2008), and the function of predicted coding protein sequences (CDSs) (mainly involved in sulfur oxidation) were identified by comparing against the NCBI non-redundant (NR) database (Pruitt et al., 2007) and Uniprot (SwissProt and TrEMBL) (Apweiler et al., 2004) using BLASTP with a minimum 40% identity and 70% coverage. Further functional information was obtained using functional domain search by the HMMER program against Pfam database (Finn et al., 2014). COG annotation was performed with rpsBlast (E-value <10^−3^) searching against the corresponding database (Roman et al., 2000). Sequences of all the protein-coding genes were uploaded to the KEGG database for the annotation of metabolic pathways (Kanehisa et al., 2004). Annotations were mainly based on the results from NCBI and Uniprot when in the case of conflicts. Complete genome sequence of *H. titanicae* SOB56 was submitted to NCBI (https://www.ncbi.nlm.nih.gov/), and the GenBank BioProject ID is PRJNA644742.

### Genomic Comparison of *Halomonas* Genomes

At the time of analysis (December 2019) the complete genome sequences of 14 *Halomonas* were downloaded from the NCBI database. The CDSs of all the genomes were predicted accordingly using the RAST Server pipeline. The average nucleotide identities (ANIs) between genomes were calculated with JSpeciesWS (Richter et al., 2016) based on BLAST+. GET_HOMOLOGUES v3.0.3 (Contreras-Moreira and Vinuesa, 2013), which included three different clustering algorithms, the Bidirectional best hit (BDBH), COGtriangles (Kristensen et al., 2010), and OrthoMCL (Li et al., 2003) algorithms, was used to cluster orthologous genes and identify the core- and pan-genome with default settings. MAFFT (Kazutaka et al., 2002) was used for multiple sequence alignment, and TrimAl v1.2 (Capella-Gutierrez et al., 2009) was used to prune the alignment results with the parameter “-gt 1.” IQ-TREE (Minh et al., 2020) was used to predict the best nucleotide substitution model for the phylogenetic tree, and core genome tree was constructed using RAxML v8.2.4 (Stamatakis, 2006) based on the maximum-likelihood (ML) algorithm (Gamma Distribution, DAYHOFF Model) with 1,000 bootstrap replicates. Homologous sequences that are closely related to TsdBA were aligned using BLAST (Altschul et al., 1997) with a minimum 40% identity and 70% coverage. ProtParam (Gasteiger et al., 2005) and TMHMM Server v2.0 (Krogh et al., 2001) were used to predict molecular weight and transmembrane structure of the predicted protein TsdBA, respectively. The signal peptide sites of TsdBA were predicted by using the SignalP v5.0 (Petersen et al., 2011). The reviewed thiosulfate dehydrogenase sequences, which were closely related with TsdBA from the Uniprot database (Apweiler et al., 2004), were selected for multiple alignments and further phylogenetic analysis. In addition, the description files of crystal structures about TsdA from *Allochromatium vinosum* 180 (PDB code: 4WQ7) and TsdB from *Marichromatium purpuratum* 984 (PDB code: 5LO9), were obtained from PDB database (https://www.rcsb.org/) for further multiple alignment. The multiple alignment of protein sequences from different strains was carried out with ClustalX v2.0 (Larkin et al., 2007) and ESPript 3.0 (Robert and Gouet, 2014), and the phylogenetic tree was constructed using MEGA v7.0 (Kumar et al., 2016) based on the NJ algorithm with 1,000 bootstrap replicates.

### Oxidation of Thiosulfate and RT-qPCR

The growth characterization and sulfur-oxidizing ability of strain SOB56 were detected in MMT medium with and without 10 mM Na_2_S_2_O_3_ as the sulfur source. The MMT medium contained the following (L^−1^): NaCl 30 g, NH_4_Cl 0.5 g, CaCl_2_•2H_2_O 0.1 g, K_2_HPO_4_ 0.5 g, MgCl_2_ 0.4 g, trace element solution 1 ml, anhydrous sodium acetate 0.8 g, and yeast extract 0.2 g. The pH of the MMT medium was adjusted to 7.0. Bacterial growth was measured by a full wavelength spectrophotometer (Multiskan GO, Thermo) at 600 nm, and the pH was recorded by a pH meter (DELTA 320, Mettler Toledo). The degradation of Na_2_S_2_O_3_ was tested by spectrophotometric iodometric determination. Thiosulfate can be oxidized to tetrathionate in alkaline solution, and 2 mol of tetrathionate will generate 3 mol of thiosulfate when alkaline is enough (Zhang and Dreisinger, 2002). The amount of all thiosulfate was also detected by spectrophotometric iodometric method after enough alkali solution was added, and the concentration of tetrathionate was calculated according to the amount of generated thiosulfate.

Cultures of *H. titanicae* SOB56 were grown in MMT medium with and without Na_2_S_2_O_3_ (10 mM) as the sulfur source. Cells were harvested after the cell density at 600 nm (OD_600_) reached 0.3, and three independent experiments were performed. RNA was extracted using the RNA extraction kit (Omega) according to the manufacturer’s instructions. Contaminating DNA was removed using the DNAfree kit (Ambion), and the purified RNA was transcribed to yield cDNA. The quantitative real-time PCR was performed using a StepOne real-time PCR System (AB Applied Biosystems) with a total reaction mixture of 20 μl containing 250 nM primers, 10 μl of SYBR Green qPCR mix, 8.5 μl of RNase-free water, and 0.2 μl of cDNA template. The PCR was performed for 5 min at 95°C, followed by 40 cycles of 1 min at 95°C, 30 s at 50°C, and 30 s at 72°C. Each sample was run in triplicate, and cycle threshold (CT) values and melting curves of each reaction were analyzed using StepOne Software v2.2. The *recA* was used as a reference gene (Florindo et al., 2012). Calibration curves (*tsdA4151*, *tsdB4152*, and *recA*) were generated using 10-fold dilutions of *H. titanicae* SOB56 genomic DNA. The primers used for qPCR are listed in Supplementary Table S1. Relative gene expression level was analyzed using 2^−ΔΔCt^ method (ΔΔCt = ΔCt_(test)_ − ΔCt_(calibrator)_, ΔCt_(test)_ = Ct_(target, test)_ − Ct_(reference, test)_ and ΔCt_(calibrator)_ = Ct_(target, calibrator)_ − Ct_(reference, calibrator)_) (Wang et al., 2017).

### Other Characteristics Related to Survival in Hydrothermal Environments

To determine the concentration of exopolysaccharides (EPS), strain SOB56 were cultured in MB medium. Cells were harvested by centrifugation at 4°C after a 24 h of cultivation. The EPS concentration of each samples was determined using the phenol/sulfuric acid method (Tsai and Frasch, 1982) with glucose as a standard, which was measured by a full wavelength spectrophotometer (Multiskan GO, Thermo) at 485 nm (Balsanelli et al., 2014). To measure the biofilm formation of strain SOB56, cells were harvested after a 24 h of cultivation in MB medium without shaking. Then the biofilm was fixed with methanol, stained with crystal violet, and washed with 33% glacial acetic acid to release the bound dye, the final absorbance was measured at 570 nm (OD_570 nm_) using a microplate absorbance reader (Tecan Sunrise, Australia). To test anaerobic growth, bacterial cultures were cultured in ASW medium supplemented with 10 mM nitrate as an electron acceptor in an anaerobic jar filled with nitrogen and a packet of AneroPack-Anaero (Mitsubishi Gas Chemical Co.) at 28°C for at least 1 month. Cultures without nitrate were used as controls. The nitrate reduction ability was detected with Griess reagents (three technical replicates; Daniela et al., 2008). In the NaCl tolerance experiment, distilled water was used to prepare the synthetic marine ZoBell broth (per liter: 5 g peptone, 1 g yeast extract, and 0.01 g FePO_4_), and NaCl concentrations were adjusted to 0%, 0.5%, and 1–37% (w/v, at intervals of 1.0%). Growth of strain SOB56 under different concentrations of metal ions (100 μM and 1 mM Zn^2+^, Co^2+^, Hg^2+^, Cu^2+^, 50 μM, and 100 μM Ni^2+^) were examined using MB medium, and bacterial growth was measured by a full wavelength spectrophotometer (Multiskan GO, Thermo) at 600 nm. For each condition and culture described above, there were three biological replicates.

## Results

### General Genome Characteristics of *Halomonas titanicae* SOB56 and Other *Halomonas* Genomes

The 16S rRNA gene sequence similarity of strain SOB56 is 100% compared with *H. titanicae* BH1^T^, which was first obtained from the *RMS* (*Royal Mail Ship*) *Titanic* wreck site during the Akademic Keldysn expedition in 1991 (Sánchez-Porro et al., 2010). The complete genome sequence of *H. titanicae* SOB56 forms a whole circle chromosome, and it is composed of 5,279,693 bp, and the calculated G + C content is 54.6%. A total of 4,771 coding genes (CDSs), 18 rRNA genes (six 5S rRNA, six 16S rRNA, and six 23S rRNA) and 60 tRNA genes are identified in the genome of *H. titanicae* SOB56 (Table 1). Among the predicted genes, 4,452 CDSs are assigned to 22 different clusters of orthologous groups (COGs), while 2,555 CDSs are annotated in the KEGG database.

**Table 1:** Genome features of *Halomonas titanicae* SOB56.

	Contigs	Total length (bp)	G + C content (%)	Gene numbers (total)	CDSs (total)	rRNAs	tRNAs
*H. titanicae* SOB56	1	5,279,693	54.6	4,853	4,771	18	60

At the time of writing, 228 genomes of the genus *Halomonas* have been sequenced and deposited in the NCBI database (Supplementary Table S2), and 14 other complete genomes from this genus are selected for further analysis. Most of these *Halomonas* genomes were obtained from salt lakes and wastewater (Supplementary Table S3). The genome sizes of the 14 related genomes ranges from 3,543,891 bp to 5,327,410 bp. The G + C contents range from 52.1% to 68.4%. The numbers of genes and proteins range from 3,321 to 4,875 and 3,231 to 4,792, respectively.

### Phylogenetic and Functional Properties of 15 *Halomonas* Genomes

**Figure 1:**
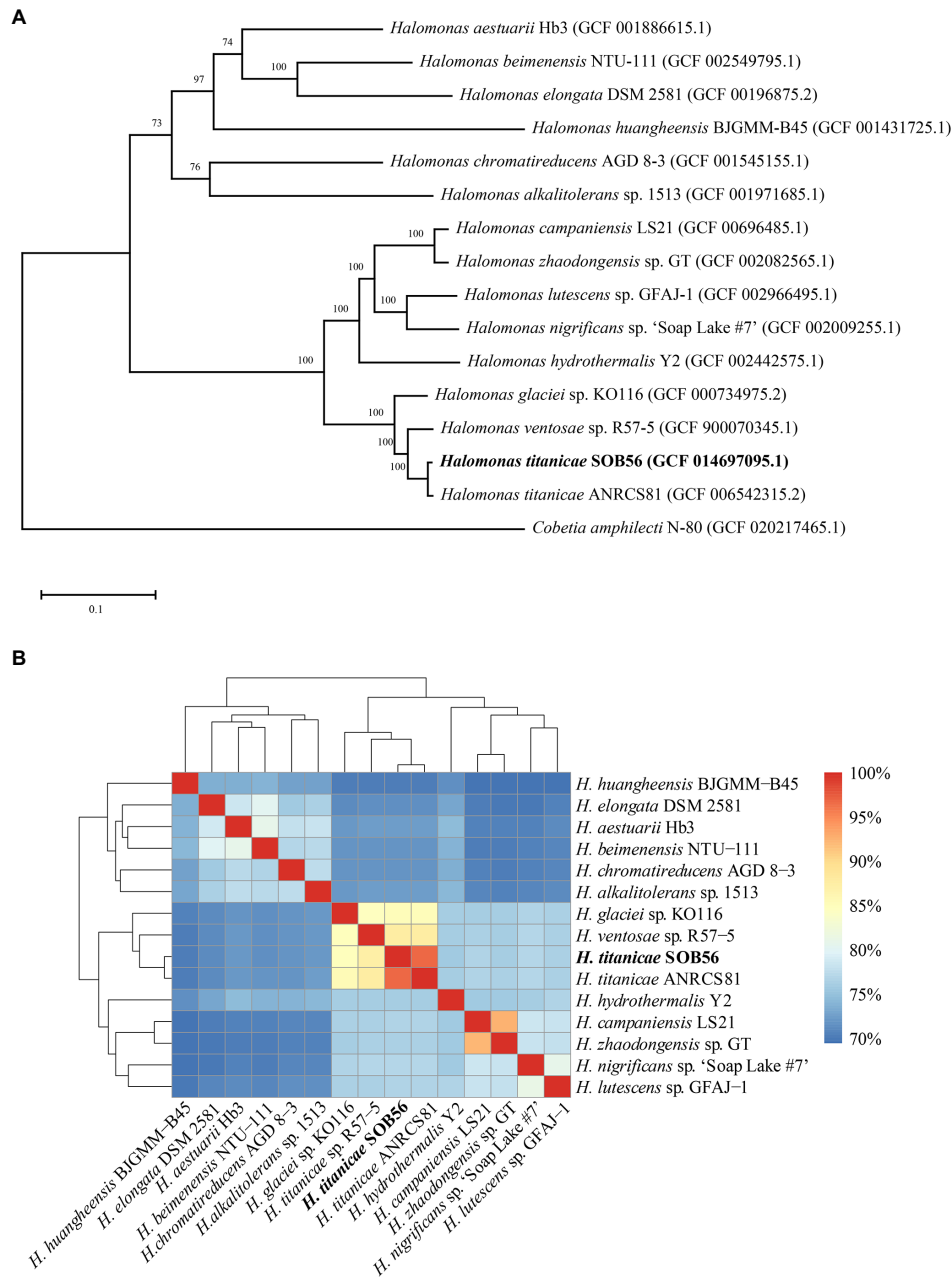
The phylogenetic relationship of 15 complete *Halomonas* genomes. **(A)** The ML tree constructed from single-copy orthologous genes of *Halomonas*. Percentage bootstrap values above 50% (1,000 replicates) are shown at branch nodes, and the scale bar indicates the number of substitutions per site. **(B)** The ANI values among these *Halomonas* genomes. *Cobetia amphilecti* N-80 is used as outgroup.

To investigate the phylogenetic relationships of the 15 *Halomonas* genomes, a phylogenetic tree was constructed based on the single-copy core genes (Figure 1A). In total, 15 genomes were divided into two clades in the core genome tree, *H. titanicae* SOB56 was most closely related to *H. titanicae* ANRCS81 and was clustered with eight other *Halomonas* genomes. The ANI values calculated through genomic pairwise comparisons further confirmed their genetic relatedness. The ANI values of the genomes in the clade including *H. titanicae* SOB56 ranged from 75.98% to 96.77%, which were higher than those of other *Halomonas* genomes (Figure 1B).

According to the KEGG annotation, the 15 *Halomonas* genomes shared many common characteristics regarding to central metabolism and energy metabolism, such as the TCA cycle, glycolysis, carbohydrate, and nitrogen metabolism (Supplementary Figure S1). Genes related to dissimilatory nitrate reduction, nitrite oxidation, and nitrous oxide reduction existed in *H. titanicae* SOB56, including genes encoding for nitrate reductase, nitrite reductase, and nitrous oxide reductase, which lead to the conversion of nitrate to nitrite, nitrite to ammonia, and nitrous oxide to nitrogen, respectively. According to the results of the core-genome phylogenetic tree and ANI values, *H. titanicae* SOB56, *H. titanicae* ANRCS81, *H. ventosae* sp. R57-5, and *H. glaciei* sp. KO116 shared 2,871 common core genes and over 84% ANI similarity. Therefore, they were selected for further COG annotation and comparison. The whole-genome COG annotation results indicated that COG R (general function prediction only) and E (amino acid transport and metabolism) accounted for a large proportion of the genomes of all four organisms (Supplementary Figure S2A). Nevertheless, the COG annotation results for species specific genes showed considerable differences in COG functional categories (Supplementary Figure S2B), which might be correlated with their diverse phylogeny and trophic strategies. In addition, *H. titanicae* SOB56 and ANRCS81 have 321 and 334 species-specific genes, respectively, and these genes have similar relative abundance in COG categories since the high genetic relationship between the two strains.

### Growth of *Halomonas titanicae* SOB56 in Thiosulfate-Containing Substrates

**Figure 2:**
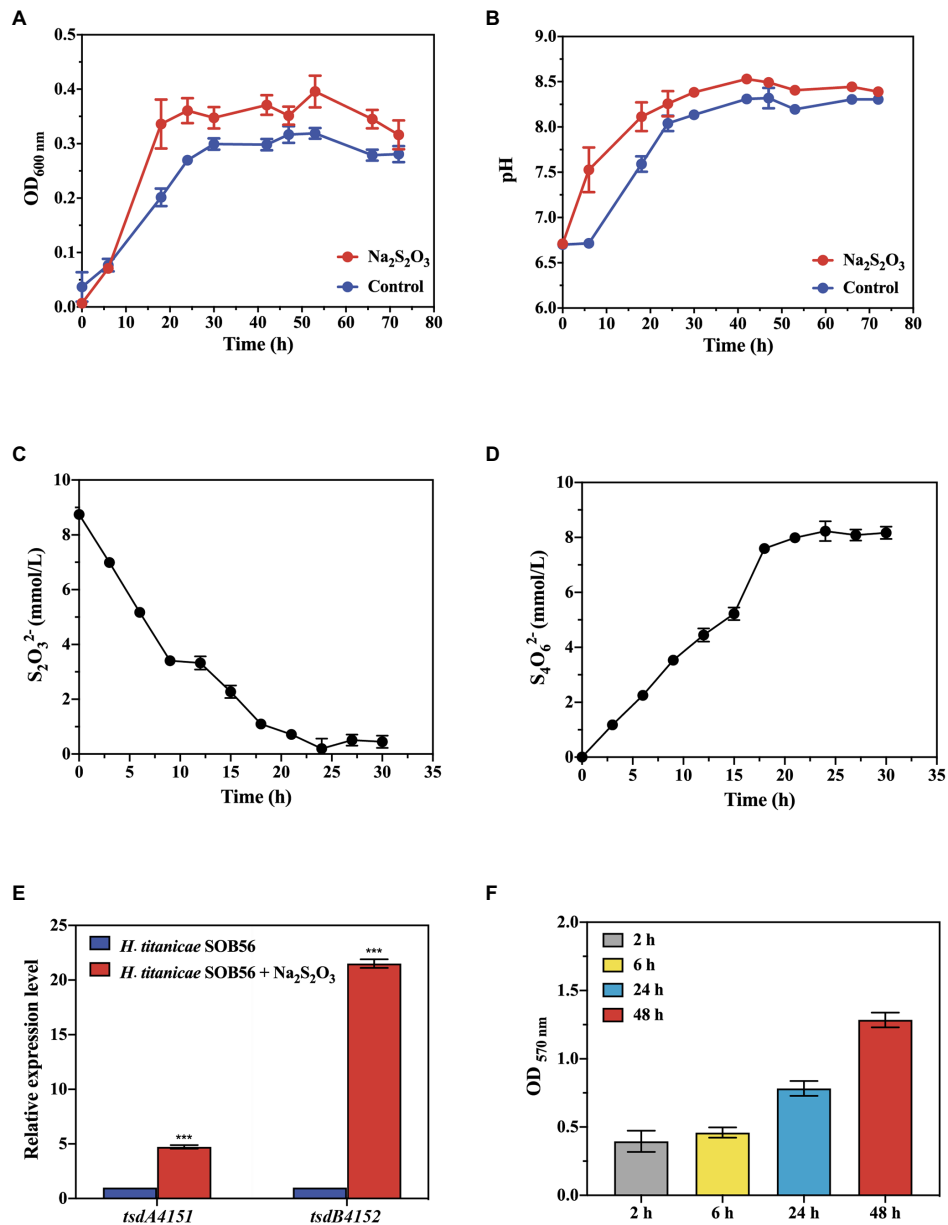
The growth of train SOB56 and its Na_2_S_2_O_3_ oxidizing ability. **(A)** The change of OD_600 nm_. **(B)** The change of pH. **(C)** The change of S_2_O_3_^2−^ concentration. **(D)** The change of S_4_O_6_^2−^ concentration. **(E)** Expression of genes *tsdA4151* and *tsdB4152* in *Halomonas titanicae* SOB56. Three biological replicates and three technical replicates were used for each gene/condition. The error bars represent the standard deviations for three replicates. For statistical analysis, significance was analyzed with a *t*-test of unpaired unequal variance, ***p < 0.01. The error bars represent the standard deviations for three replicates. **(F)** Biofilm formation of *H. titanicae* SOB56.

The growth curve and thiosulfate degradation activity of strain SOB56 were detected using spectrophotometry and spectrophotometric iodometric determination, respectively. After approximately 18 h of cultivation, strain SOB56 entered the stationary stage (Figure 2A). During the cultivation process, the pH value of the culture increased during the first 20 h and then stabilized, and the final pH was increased by 1.68 units from the initial pH (Figure 2B). In addition, the endpoint concentration of thiosulfate sodium was only 0.45 mM (Figure 2C) and over 94.86% thiosulfate was degraded. Meanwhile, it can be found that tetrathionate was accumulated while the thiosulfate was degraded (Figure 2D), and the concentration of tetrathionate reached 8.23 mM after 24 h of cultivation.

### Identification and Expression Levels of Thiosulfate Dehydrogenase-Encoding Genes *tsdBA* in *Halomonas titanicae* SOB56

In the genome of *H. titanica* SOB56, TsdA4151 was predicted a *c*-type cytochrome containing thiosulfate dehydrogenase enzyme and TsdB4152 was a *c_4_*-type cytochrome protein containing redox partner via local BLAST and phylogenetic analysis (Table 2; Supplementary Figure S3). TsdA4151 and TsdB4152 were composed of 338 and 221 amino acids and showed predicted molecular weights of 37.82 kDa and 23.50 kDa, respectively. The first 29 amino acid residues of TsdA4151 constituted a typical Sec-dependent leader peptide, including a transmembrane domain, while the first 24 amino acids of TsdB4152 constitute a signal peptide. Phylogenetic analysis showed that TsdA4151 of *H. titanica* SOB56 was clustered with TsdA from *Pseudomonas stuzeri*, *Thiomonas intermedia*, *Psychrobacter arcticus*, and *A. vinosum*. TsdB4152 was related to thiosulfate dehydrogenase electron acceptor TsdB from *P. stuzeri* and *T. intermedia* (Supplementary Figure S3). Therefore, TsdA4151 and TsdB4152 may contribute to the thiosulfate oxidation pathway in *H. titanicae* SOB56. Furthermore, by searching for genes involved in sulfur metabolism, the homologous genes encoding for Sox complex (SoxXYZABCDEFG), thiosulfate:quinone oxidoreductase (DoxDA) and sulfite dehydrogenase (SoeABC) were absent in SOB56 genome.

**Table 2:** Homologous proteins of TsdA in *Halomonas* genomes.

Name	Number and annotation function	TsdA reference proteins (Uniprot ID)	Corresponding species	E-value	Coverage	Identity
*H. titanicae* SOB56	TsdA4151; cytochrome *c* family	sp|D5WYQ5|TSDA_THIK1	*T. intermedia* K12	5.82E-81	0.92	0.403
*H. aestuarii* Hb3	WP_071942102.1 cytochrome *c*	sp|A4VND8|TSDA_PSEU5	*P. stutzeri* A1501	1.6E-115	0.92	0.537
*H. campaniensis* LS21	WP_038486983.1 cytochrome *c*	sp|D5WYQ5|TSDA_THIK1	*T. intermedia* K12	4.84E-82	0.77	0.455
*H. ventosae* sp. R57-5	WP_050713698.1 MULTISPECIES: cytochrome *c*	sp|D5WYQ5|TSDA_THIK1	*T. intermedia* K12	1.57E-81	0.92	0.404
*H. titanicae* ANRCS81	CP039374.2 cytochrome *c*	sp|D5WYQ5|TSDA_THIK1	*T. intermedia* K12	1E-84	0.97	0.401
*H. lutescens* sp. GFAJ-1	WP_009098701.1 cytochrome *c*	sp|D5WYQ5|TSDA_THIK1	*T. intermedia* K12	1.87E-82	0.72	0.458
*H. zhaodongensis* sp. GT	WP_083006270.1 cytochrome *c*	sp|D5WYQ5|TSDA_THIK1	*T. intermedia* K12	5E-82	0.76	0.455
*H. glaciei* sp. KO116	WP_035555383.1 cytochrome *c*	sp|D5WYQ5|TSDA_THIK1	*T. intermedia* K12	7.99E-83	0.76	0.457
*H. hydrothermalis* Y2	WP_096923463.1 cytochrome *c*	sp|D5WYQ5|TSDA_THIK1	*T. intermedia* K12	3.13E-82	0.75	0.448

The expression levels of genes *tsdA4151* and *tsdB4152* in strain SOB56 were detected using RT-qPCR under cultivation conditions with or without the addition of Na_2_S_2_O_3_. The relative expression levels of *tsdA4151* and *tsdB4152* during cultivation with Na_2_S_2_O_3_ were significantly upregulated (4.3 times and 21.5 times, respectively, *p* < 0.01) (Figure 2E). In addition, the expression level of *tsdB4152* was much higher than that of *tsdA4151.* This result indicated that both genes were involved in the metabolism of Na_2_S_2_O_3_ in strain SOB56.

### Distribution and Characteristics of TsdA- and TsdB-Encoding Genes in 15 *Halomonas* Genomes

The homologous proteins of TsdA were found in nine *Halomonas* genomes (Table 2), and all of them were annotated as cytochrome *c* family proteins. The amino acid sequence identities among these TsdA proteins ranged from 40.1% to 53.7%. The molecular weights of these nine proteins ranged from 35.52 to 39.09 kDa. Moreover, a gene encoding thiosulfate dehydrogenase electron acceptor (TsdB) was located immediately upstream of the *tsdA* gene in each of the nine *Halomonas* genomes.

**Figure 3:**
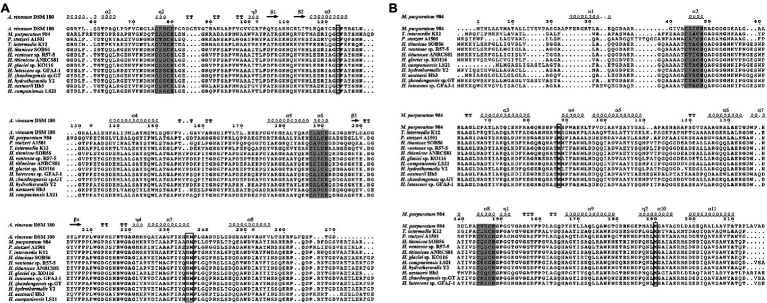
Multiple sequence alignment of the TsdA **(A)** and TsdB **(B)** in *Halomonas titanicae* SOB56 and related organisms. Protein secondary structures were shown on the top of sequences. Referred crystal structures of TsdA and TsdB were from *Allochromatium vinosum* 180 (PDB code: 4WQ7) and *Marichromatium purpuratum* 984 (PDB code: 5LO9), respectively. Heme-binding motifs are indicated by gray boxes, and putative distal heme ligands are marked by black squares. Strictly conserved residues are marked with bold font in sequences.

TsdA and TsdB sequences from the nine *Halomonas* strains and related bacteria were analyzed via multiple alignments (Figure 3). Two typical conserved regions containing a cysteine (Cys)-X2-cysteine (Cys)-histidine (His) sequence, representing a typical heme c binding site, were found in all TsdA and TsdB sequences. The two hemes in TsdA are covalently bound sites to the polypeptide chain through thioether bonds formed by cysteine residues Cys_76_ and Cys_79_ for the first heme and Cys_187_ and Cys_190_ for the second heme. Sequence alignment of various TsdA proteins (Figure 3A) reveals a conserved Cys_123_ close to the first heme. The first heme iron is hexacoordinated with His_80_ as the proximal axial ligand and Cys_123_ as the distal one (Brito et al., 2015). In TsdA of *A. vinosum*, the second heme iron is axially coordinated by His_191_ and Lys_235_ (Brito et al., 2015), but in *M. purpuratum* 984, *T. intermedia* K12 and all *Halomonas* species, the Lys_235_ equivalent position is asparagine (Asn), and in *Pseudomonas stutzeri* A1501 is replaced by serine (Ser). Remarkably, a conserved Met_236_ is followed in all TsdA proteins. Furthermore, the secondary structure indicates these TsdA proteins harbor eight helices in total, and every four helices surround each heme molecule (Figure 3A). In a number of microorganisms, for example, *T. intermedia*, *P. stutzeri*, *M. purpuratum*, *Sideroxydans lithotrophicus*, *Thiocystis violascens*, and *Thiorhodococcus drewsiithe*, *tsdA* gene is immediately preceded by *tsdB* encoding for another diheme cytochrome (Denkmann et al., 2012; Kurth et al., 2016), and the same character is also observed in *Halomonas*. The alignment of various TsdB proteins (Figure 3B) reveals that two hemes are formed by cysteine residues Cys_44_ and Cys_47_ for the first heme, and Cys_144_ and Cys_147_ for the second one. Two conserved methionine residues but no conserved histidines or cysteines were identified in these TsdB proteins, which indicate both of hemes have axial coordination by His/Met.

**Figure 4:**
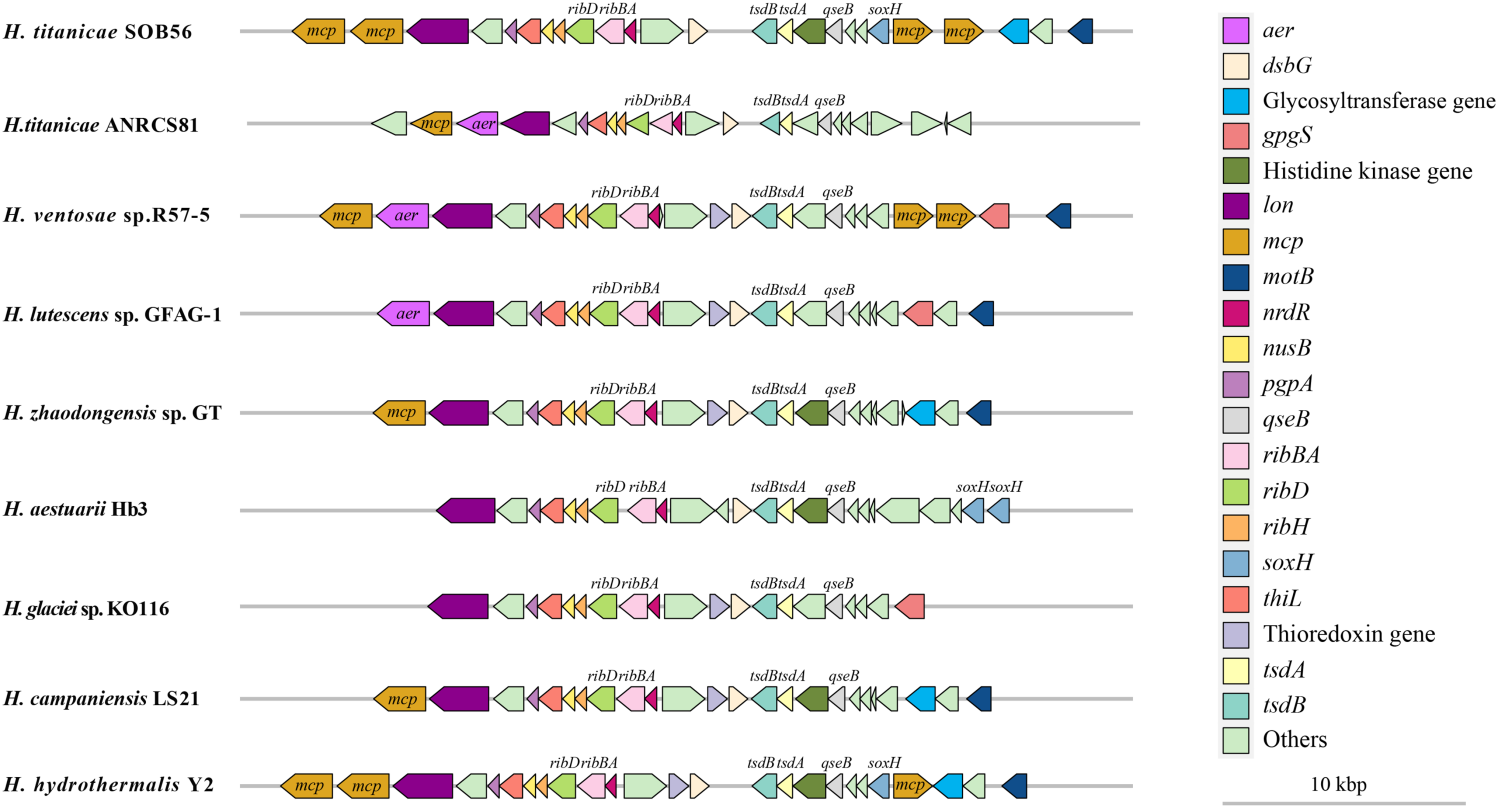
Distribution of *tsdA*, *tsdB*, and its adjacent genes in *Halomonas* genomes.

The arrangement of upstream and downstream genes of *tsdA* and *tsdB* in the nine *Halomonas* genomes was also analyzed. A common upstream gene of *tsdB* was a gene encoding the two-component system regulator QseB, while the downstream genes of *tsdA* included genes encoding the thiol: disulfide interchange protein DsbG, three riboflavin metabolism-related proteins (RibH, RibBA, and RibD), the transcriptional termination protein NusB, and two thiamine-monophosphate kinases (EC 2.7.4.16) (Figure 4). A gene encoding a signal transduction-related histidine kinase was predicted to be located next to *qseB* in *H. titanicae* SOB56 as well as in four other *Halomonas* genomes, and a thioredoxin-encoding gene was found adjacent to DsbG in six *Halomonas* genomes, except for *H. titanicae* SOB 56, *H. titanicae* ANRCS81, and *H. aestuarii* Hb3. Moreover, four genes encoding methyl receptor chemotaxis proteins (MCPs) were predicted near *tsdA* and *tsdB* in *H. titanicae* SOB56, which might play important roles in sensing and responding to the hydrothermal environment.

### Other Characteristics of Sulfur Metabolism in *Halomonas titanicae* SOB56

**Figure 5:**
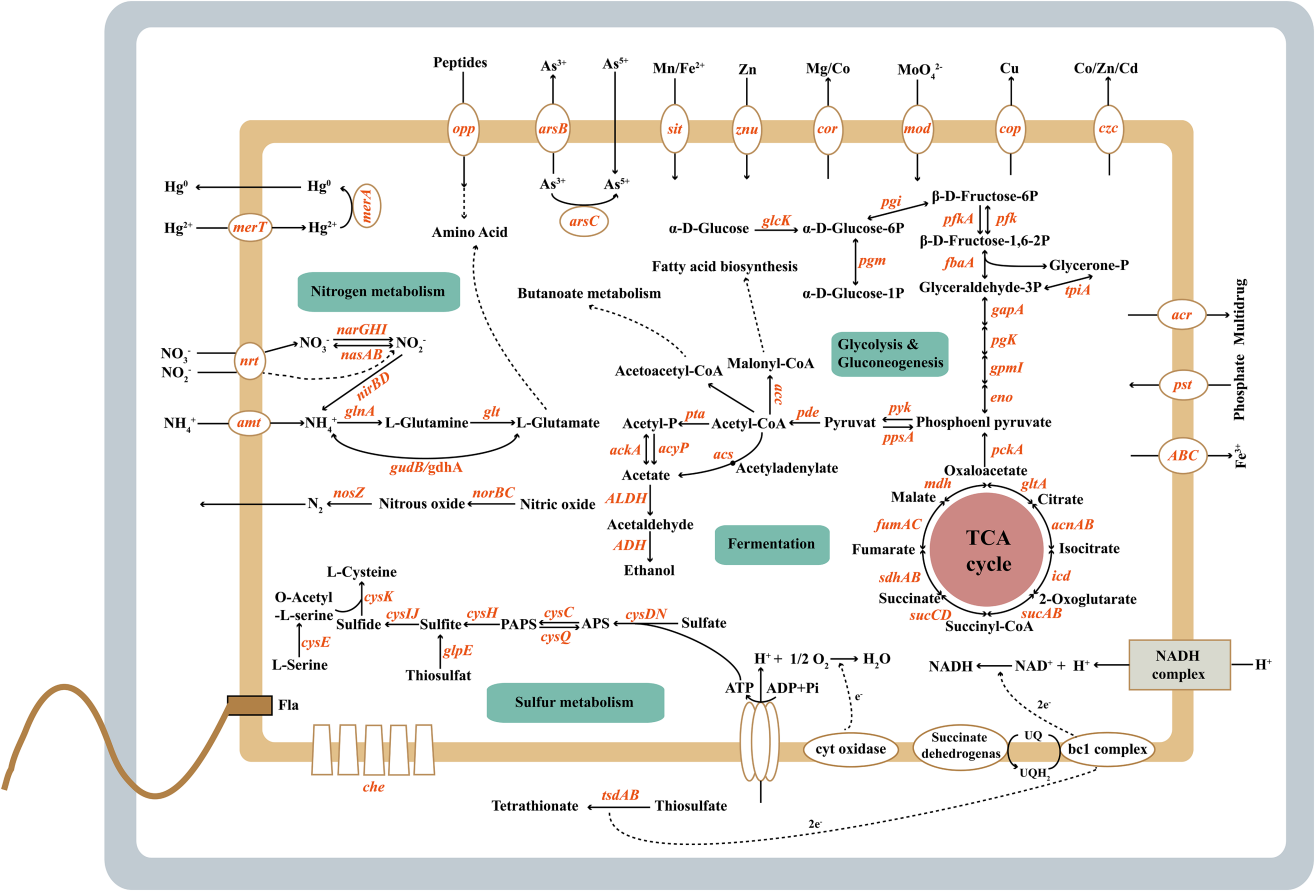
The metabolic features of *Halomonas titanicae* SOB56.

Genes involved in assimilatory sulfate reduction were present in *H. titanicae* SOB56 (Figure 5). Sulfate uptake was performed by the sulfate and thiosulfate ABC transporter CysAWTP or by two different kinds of sulfate permeases. One sulfate permease belonged to the high-affinity transporter SulP, and the other one was a hypothetical sulfate permease (CysZ). In *H. titanicae* SOB56, the whole metabolic process from sulfate to cysteine was identified, in which sulfate is gradually reduced to sulfite by adenylyltransferase (CysDN), adenylylsulfate kinase CysC, 3′(2′), 5′-bisphosphate nucleotidase CysQ and APS reductase (CysH). An assimilatory sulfite reductase (CysIJ) and cysteine synthase (CysK) were also identified, which reduce sulfite to sulfide and catalyze the synthesis of cysteine from O-acetylserine and sulfide, respectively (Kredich, 1992; Sievert et al., 2008). Moreover, *H. titanicae* SOB56 has a gene encoding rhodanese (EC 28.1.1), which catalyzes the conversion of thiosulfate to sulfite and performs thiosulfate assimilation (Adams et al., 2002). The gene encoding for rhodanese is one of the 321 specific genes observed in *H. titanicae* SOB56 through COG strain-specific gene annotation, which was not identified among other *Halomonas* genomes.

In addition, the co-clustering of genes encoding components of the DsrMKJOP sulfite reduction complex was identified in *H. titanicae* SOB56, these proteins may mainly function in the transport of sulfur atoms because of the absence of DsrAB. Three genes encoding DsrEFH, which belonging to the DsrMKJOP gene cluster, were also found in strain SOB56. DsrE, DsrF, and DsrH were homologs of TusD, TusC, and TusB in the sulfur relay system and were involved in the biosynthesis of 2-thiouridine. Furthermore, IscS and TusE were present, IscS specifically transfers sulfur to DsrE (Cort et al., 2008), while TusE binds the TusBCD complex and stimulates sulfur transfer from TusA to TusD (Ikeuchi et al., 2006). Homologs of TusBCD and TusE were mainly found in *Gammaproteobacteria* as well as in some phototrophic bacteria with a large gene cluster responsible for sulfur oxidation (Andrea and Christiane, 1998; Dahl et al., 2005).

### Carbon and Nitrogen Metabolism and Respiration of *Halomonas titanicae* SOB56

In *H. titanicae* SOB56, a series of genes involved in carbon metabolism account for a relatively higher abundance than genes involved in other metabolic pathways, and crucial central metabolism pathways are present, for example, glycolysis and the tricarboxylic acid cycle. Genes encoding for key enzymes involved in amino sugar and nucleotide sugar metabolism, organic acid conversion, and pentose phosphate pathway were also identified. Strain *H. titanicae* SOB56 could form visible colonies on the agar plate, and the nitrate reduction test was positive with Griess reagents under the anaerobic condition. Similarly, genes encoding membrane-associated nitrate reductase (NarGHI) and cytoplasmic assimilatory nitrite reductase (NirBD) (Doroshchuk et al., 2006), which could catalyze the conversion of nitrate to nitrite and nitrite to ammonia, were present in the genome. Two pathways of ammonium assimilation and glutamate biosynthesis were identified, and the key catalytic enzymes were NADP-linked glutamate dehydrogenase and glutamine synthetase (Reitzer, 2003; Muro-Pastor et al., 2005; Suzuki and Knaff, 2005; Leigh and Dodsworth, 2007). In addition, strain SOB56 possesses nitric oxide reductase (NorBC) and the nitrous oxide reductase complex (Nos), which catalyze the conversion of nitric oxide to nitrous oxide and nitrous oxide to nitrogen, respectively. The essential enzymes for denitrification, such as nitrite reductases (NirK or NirS), are absent in *H. titanicae* SOB56 (Figure 5). Urease-coding genes *ureABCDEFG* that catalyze the degradation of urea to ammonia and carbamide acid were found in the genome. In addition, there are more than 400 genes related to amino acids and derivatives metabolisms and ammonia ion assimilation, which enable the bacterium to utilize various organic nitrogen effectively.

Regarding aerobic respiration, *H. titanicae* SOB56 contained genes encoding complexes I-IV of the respiratory chain, including NADH ubiquinone oxidoreductase (NuoABCDEF, complex I), succinate dehydrogenase (SdhABCD and FrdA, complex II), the cytochrome *bc_1_* complex (PetAB, complex III), and cytochrome *c* oxidoreductase (CcoNOPQ, complex IV). Additionally, it exhibited a *bd*-type cytochrome oxidase (CydAB) and low oxygen adapted *cbb_3_*-type cytochrome *c* oxidase. The presence of the *atpABCDEFGH* genes in its genome indicates that the respiratory chain is linked to an F0F1 ATPase that generates ATP.

### Motility, Chemotaxis, Biofilm, and Exopolysaccharide Production

The genome of *H. Titanicae* SOB56 comprised 48 genes involved in flagellar assembly, including the complete set of genes for flagellar biosynthesis and flagellar structure proteins (Figure 5). This was consistent with a previous study showing that *H. titanicae* BH1 exhibits motility via peritrichous flagella (Sánchez-Porro et al., 2010). Transcriptional regulators of flagellar synthesis were also identified in the genome, such as Flgm, Flia (σ^28^), Flhc, and Flhd. In addition, genes encoding chemotaxis proteins related to motility, such as CheAWRBYZ and methyl-accepting chemotaxis protein (MCP), were present.

*H. titanicae* SOB56 could form biofilms in MB medium, and the amount of biofilm increased continuously within 48 h (Figure 2F). A homologous gene cluster of the Wsp chemosensory system was present in its genome, and this cluster included WspA, WspE, and WspR, which might be involved in the regulation of biofilm formation (Hickman et al., 2005). Furthermore, strain SOB56 processed a large array of genes related to the synthesis and export of exopolysaccharides (EPS), including genes encoding exopolysaccharide synthase and glycosyl transferases. EPS (0.4 mg ml^−1^) from *H. titanicae* SOB56 were detected after cultivation in MB medium for 24 h. It could be inferred that the synthesized EPSs might help *H. titanicae* SOB56 increase its substrate uptake rate by attaching to organic material in hydrothermal sediment. Moreover, 40 CDSs encoding GGDEF (diguanylate cyclase), EAL (diguanylate phosphodiesterase), and PAS (Per-Arnt-Sim) domains were found in the genome, which have been proven to play key roles in biofilm formation and motility.

### Metal Resistance and Osmotic Stress Regulation of *Halomonas titanicae* SOB56

The genome of *H. titanicae* SOB56 contained various genes related to heavy metal resistance and efflux systems (Supplementary Table S4; Figure 5), including two genes encoding heavy metal translocating P-type ATPases, which may participate in the transport of Pb^2+^, Cd^2+^, Zn^2+^, Hg^2+^, and Cu^2+^ against the concentration gradient to the periplasm (Nies, 1999). Zn^2+^, Co^2+^, Cu^2+^, Cd^2+^, and Ni^2+^ might be transported by RND family efflux transporter proteins from both the cytoplasm and the periplasm to the outside of the cell (Nies, 1999; Choudhury and Srivastava, 2001). P-type ATPases are usually regulated by MerR family regulators responding to the intracellular heavy metal concentration (Choudhury and Srivastava, 2001; Brown et al., 2003), and four homologs of MerR family regulators were found in *H. titanicae* SOB56. Three mercuric transport proteins (MerE, MerC, and MerT) for organic mercury uptake (Sone et al., 2010) were also present. In addition, two mercuric-resistance operon regulatory proteins and one mercuric ion reductase (MerA), which can catalyze the transformation of Hg^2+^ to Hg^0^ (Felske et al., 2003), were identified in the genome. Simultaneously, strain SOB56 could grow in the MB medium with 100 μM and 1 mM Zn^2+^, Co^2+^, Cu^2+^, and 50 μM and 100 μM Ni^2+^ (Supplementary Figure S4). However, 100 μM Hg^2+^ inhibited the growth of strain SOB56 that may due to the relatively high concentration. These shreds of evidence might contribute to the strategies available for *H. titanicae* SOB56 to adapt metal-rich deep-sea hydrothermal environments.

Bacteria in the genus *Halomonas* are typical moderate halophiles showing a wide range of salinity adaptability. Therefore, the growth of strain SOB56 under different Na^+^ concentrations showed the ability that could grow in the range of 0%–24% (w/v) NaCl concentrations (Supplementary Figure S5). In total, 24 genes in t *H. titanicae* SOB56 were found to be involved in choline and betaine uptake and biosynthesis. Betaine can be synthesized by choline dehydrogenase (EC 1.1.99.1), which is a membrane-bound oxygen-dependent enzyme, possibly in combination with betaine aldehyde dehydrogenase (EC 1.2.1.8) (Wargo et al., 2008). A common strategy of osmo-adaptation in bacteria is the accumulation of compatible solutes (Kuhlmann and Bremer, 2002), such as ectoine. *H. titanicae* SOB56 contained five genes involved in the biosynthesis and regulation of ectoine, including EctABCD. It was indicated that it might be capable of accumulating ectoine and its hydroxylated derivative hydroxy-ectoine (García-Estepa et al., 2006). Osmo-regulated periplasmic glucan (OPG) concentrations in the periplasm have been reported to increase in response to a decrease in environmental osmolarity (Bohin, 2000). There are five genes in strain SOB56 that are involved in the synthesis of osmo-regulated periplasmic glucans, including glucan biosynthesis glucosyltransferase and glucan biosynthesis protein-encoding genes.

## Discussion

Although it is documented that chemoautotrophic sulfur-oxidizing bacteria are the primary producers in deep-sea hydrothermal systems, heterotrophic sulfur-oxidizing bacteria may also play important roles in sulfur cycling in hydrothermal vents. Bacteria of the genus *Halomonas* have been isolated from many different water and soil environments, mainly from saline, hypersaline, or alkaline habits, including hydrothermal vents (Kaye et al., 2004; Vetriani et al., 2005; Simon-Colin et al., 2008). It was reported that the abundance of *Halomonas* bacteria in hydrothermal fluids collected from the Pacific Ocean was estimated to account for up to 28% of the total microorganisms (Kaye and Baross, 2000).

Many previous studies of the genus *Halomonas* have concentrated on osmoadaptation and exopolysaccharide production by these bacteria, whereas their roles in marine sulfur and carbon cycle have rarely been studied. In total, 11 of 13 identified culturable marine haloalkaliphilic isolates from *Gammaproteobacteria* that could incompletely oxidize sulfur compounds to tetrathionate were affiliated with *Halomonas* (Sorokin, 2003). Recently, Mandal et al. (Mandal et al., 2020). found that the genus *Halomonas*, which could form tetrathionate from the oxidation of thiosulfate by TsdA, was one of the major marine bacteria (accounting for 3%–7.8% of metagenomic reads) in the sediment of India’s west coast and influenced the anoxic sedimentary sulfur cycle (Ghosh et al., 2020). However, the biogeochemical role of this genus and its adaptive strategies in extreme marine habitats was unclear. *H. titanicae* SOB56 was isolated from the sediment of the Tangyin hydrothermal field in the southern part of the Okinawa Trough. In this study, we shed light on the potential functions of heterotrophic *Halomonas* bacteria in hydrothermal sulfur cycle and describe survival advantages of *H. titanicae* SOB56 in deep-sea hydrothermal field.

### Sulfur Metabolism Properties of *Halomonas titanicae* SOB56

Thiosulfate-oxidizing heterotrophs are widespread, particularly in the extreme marine environments due to their eco-physiological flexibility. *Halomonas* is one of the most frequent genera observed in marine environments using traditional culture methods (Sinha et al., 2019). In our study, *H. titanicae* SOB56 could grow well in MMT medium supplemented with thiosulfate sodium and was a typical alkali-producing sulfur-oxidizing bacteria. Recent study supports our observation where it has shown that *Halomonas* species could aerobically oxidize sulfide and thiosulfate (Wang and Shao, 2021). Furthermore, the formation of tetrathionate from the oxidation of thiosulfate is widespread among prokaryotes, some species of *Halomonas* also possess this physiological trait, and our efforts on the detection of tetrathionate production (Figure 2D) also add powerful proof.

It has been reported that TsdA is the key enzyme catalyzing the tetrathionate-forming reaction in many bacteria (Hensen et al., 2006; Denkmann et al., 2012; Brito et al., 2015; Pyne et al., 2018; Rameez et al., 2020). In *H. titanicae* SOB56, two potential thiosulfate dehydrogenase-encoding genes (*tsdA4151* and *tsdB4152*) were identified, and the results of RT-qPCR implied an important role of these two genes in thiosulfate oxidation. Notably, there was a big difference in the expression levels between *tsdA* and *tsdB*. It was reported that TsdB is not itself reactive with thiosulfate oxidation but accepts electrons even when TsdA and TsdB do not originate from the same source organism (Denkmann et al., 2012). Meanwhile, the electron acceptor of TsdA was distinct in different source organisms (Denkmann et al., 2012; Dahl, 2017). Thus, the higher expression of TsdB suggests that it might not be the exclusive electron acceptor of this pathway and could also accept other electrons. According to the phylogenetic tree of thiosulfate dehydrogenase and its redox partner (Supplementary Figure S3), the TsdA4151 in *H. titanicae* SOB56 was clustered with the TsdA from *T. intermedia*, which exhibits the highest activity at pH 3.0 and almost loses its activity at pH 7.0 (Denkmann et al., 2012). Furthermore, it was noteworthy that TsdA in strain *Paracoccus thiocyanatus* had a higher substrate-affinity than the Sox mechanism (Rameez et al., 2020). Therefore, the widespread presence of sulfur oxidation pathways is one of the most critical reason that heterotrophic bacteria could thrive in extreme environments, such as *Halomonas* (Wang and Shao, 2021) and *Erythrobacter flavus* (Zhang et al., 2020).

According to the previous reports about TsdA (Denkmann et al., 2012; Brito et al., 2015), the two strictly conserved cysteines in the typical Cys-X2-Cys-His heme-binding motifs of *c*-type cytochromes (similar to TsdA of the purple sulfur bacterium *A. vinosum*), provide a thioether linkage to heme, and the adjacent histidine serves as the fifth ligand for heme. The site-directed mutagenesis of Cys_123_ in the TsdA protein (Denkmann et al., 2012) and TsdA crystal structure (Brito et al., 2015) verified that Cys_123_ was the catalytic active site and the distal axial coordination of the first heme. As shown by the results of multiple sequence alignment of TsdA in genus *Halomonas*, the strictly conserved cysteine residue (Cys_123_) exists in all of the included sequences (Figure 3A). In *A. vinosum*, the distal axial ligand of the second heme switch from Lys_235_ to Met_236_ is observed upon reduction of TsdA (Brito et al., 2015). However, an asparagine residue at the Lys_235_ equivalent position is highly conserved among proteobacterial thiosulfate dehydrogenases, for example, TsdA from the *T. intermedia* and *S. lithotrophus* belonging to *Betaproteobacteria*, as well as *Campylobacter jejuni* from *Epsilonproteobacteria* (Denkmann et al., 2012; Jenner et al., 2019). In our results, all *Halomonas* species and referred *M. purpuratum*, from the same class as *A. vinosum* belonging to *Gammaproteobacteria*, possess TsdA homologues with an Asn residue instead of Lys. As reviewed TsdA containing conserved Asn_235_, the distal axial coordination of the second heme was Met_236_ (Denkmann et al., 2012; Brito et al., 2015; Jenner et al., 2019). Furthermore, it is reported that introducing Lys in place of Asn in the TsdA of *C. jejuni*, the second heme allows Lys to provide the distal ligand (Jenner et al., 2019). Thus, we speculated that although TsdAs in *Halomonas* contain conserved Asn_235_, the second heme is directly coordinated with Met_236_, and a ligand switch would not happen upon enzyme reduction. Based on the coordination relationship between cytochrome *c* and heme and the above results (Kurth et al., 2016), we inferred that the His_80_/Cys_123_ and His_191_/Met_236_ residues of TsdA act as potential axial ligands for hemes in *Halomonas*. Two pairs of His/Met residues in *Halomonas* TsdB homologues are also potential axial ligands for heme. When TsdA and TsdB originate from the same source organism, TsdB is the immediate electron acceptor of TsdA (Kurth et al., 2016). Meanwhile, *c_4_*-type cytochromes have been reported to donate electrons to the *cbb_3_* terminal oxidase in various oxygen-respiring bacteria (Chang et al., 2010; Arai et al., 2014; Barco et al., 2015; Kurth et al., 2016), and it is possible that TsdB could serve as a direct electron donor to *cbb_3_*-type cytochrome *c* oxidases in these tetrathionate-forming thiosulfate oxidizers, such as *Halomonas*. However, we did not successfully obtain a recombinant thiosulfate dehydrogenase TsdA enzyme using the pET-24a (+) and pET-28a (+) vectors in *Escherichia coli* BL21. TsdA has 23 amino acids belonging to the transmembrane region and may be difficult to be heterogeneously express using these vectors. Therefore, the recombinant expression of the gene in strain SOB56 still requires further exploration, and gene knockout or site-directed mutation experiments should be performed to verify its functional characteristics.

In the analysis of *tsdA*, *tsdB* and their adjacent genes in the *Halomonas* genomes (Figure 4), the upstream region of *tsdB* was found to contain genes encoding histidine kinase and QseB, which are related to the sensing of environmental signals and signal transduction. QseBC is an important regulator of quorum sensing (QS) (Sperandio et al., 2002), and a gene encoding LuxS, which is involved in autoinducer-2 (AI-2) production, was identified in *H. titanicae* SOB56. It was inferred that thiosulfate oxidation may be regulated by a QS mechanism. Although Sox gene cluster was absent, *soxH* homologue encoding genes were found near *tsdB*s in some *Halomonas* genomes, which might have some potential function in the thiosulfate oxidation. The neighboring genes of *tsdBA* also included two genes encoding MCPs containing PAS domains, which might be involved in energy taxis, in which the redox state of components of the electron transport chain is sensed by redox-sensitive MCPs. This phenomenon might contribute to bacterial motility toward microenvironments with optimal concentrations of reductants and oxidants (Alexandre et al., 2004). The distributions of *tsdA*, *tsdB*, and their adjacent genes were relatively conserved in the *Halomonas* genomes. Hydrothermal vents are characterized by extremely complex redox conditions, and the above characteristics may be beneficial in allowing species to perceive changes in various chemical signals and execute corresponding chemotaxis reactions in hydrothermal environments.

### Adaptation Properties of *Halomonas titanicae* SOB56 to Deep-Sea Hydrothermal Vents

In hydrothermal environments, a complete flagellum and chemotaxis system would provide *H. titanicae* SOB56 with a significant advantage in reaching a food source before its competitors or traveling away from substances that adversely affect central metabolic processes. Furthermore, 40 CDSs embraced GGDEF and/or EAL domains and PAS domains were found in the genome of *H. titanicae* SOB56 by searching against the Pfam database. The GGDEF and/or EAL domains were associated with diguanylate cyclase and phosphodiesterase activity, respectively, and can regulate biofilm formation, auto-aggregation, and motility (Ahmad et al., 2020). A large number of genes for sensing and responding to environments (EAL- and GGDEF-domain proteins and methyl-accepting chemotaxis proteins) were common features in some chemolithoautotrophic bacteria from genera *Hydrogenmovibrio*, *Thiomicrorhabdus* and *Thiomicrospira*, which were widespread organisms in hydrothermal environments (Scott et al., 2018). Moreover, genes that carried PAS domains might be involved in energy taxis (Scott et al., 2018), and PAS domains can play a role in the monitoring of environmental changes in light, redox potential, O_2_ or small ligands, etc. (Taylor and Zhulin, 1999). So, these CDSs may work in perceiving microenvironments with an optimal concentration of reductant and oxidant for hydrothermal microbes. Under anaerobic conditions, the presence of nitrate reductase in *H. titanicae* SOB56 was coincident with the positive phenotypic result, which means an ability that could use nitrate as an electron acceptor for anaerobic respiration. This result is also consistent with the phenotypic characteristics of some *Halomonas* species reported by Mata et al. (Mata et al., 2002). Simultaneously, the genome of *H. titanicae* SOB56 also contains the nitrite reductase NirBD. The co-existence of nitrate reductase and nitrite reductase indicates the complete dissimilatory nitrate reduction pathway (DNRA or denitrification) that reduces nitrate to ammonia. Furthermore, sulfur oxidation coupled with DNRA is usually an important source of energy for dissolved inorganic carbon fixation in hydrothermal vents (Shao et al., 2010; Li et al., 2018). In addition, various aerobic respiration complexes (complex I-IV and *cbb_3_*-type cytochrome *c* oxidase) are also present in *H. titanicae* SOB56, and these encoding genes may help the bacterium grow at a wide range of oxygen concentrations. Therefore, these pathways and features are extremely beneficial for strains who live in complicated hydrothermal environments with steep gradients of chemical factors and may also exist in other heterotrophic microbes.

Many halophiles and psychrophiles adapt to extremophile environments with high salinity or low temperature by accumulating compatible solutes, such as ectoine, glycine betaine, proline, or trehalose (Sleator and Hill, 2002; De Maayer et al., 2014). Genome analysis showed that genes involved in the synthesis or transport of these molecules were present in *H. titanicae* SOB56, which suggests that the bacterium exhibits different types of osmotic pressure regulation. A growing body of evidence has indicated that the role of compatible solutes goes beyond osmotic adjustment alone and includes the protection of cells and cell components from freezing, desiccation, high temperature, or oxygen radicals (García-Estepa et al., 2006). In addition, bacteria living in environments with high temperatures require proteins to be especially flexible (Feller, 2013) and correctly folded (Williams et al., 2011). The heat shock protein DnaK identified in *H. titanicae* SOB56 can form chaperone machinery with cochaperones DnaJ and GrpE, which participate actively in the response to hyperosmotic and heat shock conditions by preventing the aggregation of stress-denatured proteins. The existence of the DnaK gene cluster was widespread in microorganisms who thrive in hydrothermal vents, such as *Thiomicrospira* (Tang et al., 2013). In addition, *H. titanicae* SOB56 might be resistant to many metals such as lead, cadmium, zinc, and mercury, similar to *H. zincidurans* B6^T^ (Huo et al., 2014). Meanwhile, many reported *Halomonas* genomes (Wang and Shao, 2021) (including *H. titanicae* SOB56) in hydrothermal vents carry genes encoding copper resistance proteins, implying the tolerance to such bio-toxic metals in the environment.

## Conclusion

Genome analysis and laboratory experiments revealed that *H. titanicae* SOB56 was a typical alkali-producing thiosulfate-oxidizing bacterium harboring-related functional genes (*tsdA4151* and *tsdB4152*). The ability to oxidize thiosulfate enables *H. titanicae* SOB56 to utilize sulfur-containing compounds as supplementary energy resources. Compared with the other 14 complete *Halomonas* genomes, the presence of thiosulfate dehydrogenase is common in the genus *Halomonas* and shows conservative characteristics in terms of protein structure and gene locations. Moreover, the thiosulfate-oxidizing process might be regulated by QS, and the AI-2 synthesis protein LuxS was also identified in the genome of *H. titanicae* SOB56. In addition, a complete sulfate assimilation pathway is present, as are a series of genes related to adaptation to extreme environments. In summary, *H. titanicae* SOB56 might actively participate in the hydrothermal sulfur cycle and utilize sulfur compounds as an energy source. The identified characteristics related to motility, biofilm formation, heavy metal, and osmotic resistance provide the bacterium with several favorable survival strategies to cope with changeable marine environments, allowing it to occupy a wide ecological niche encompassing various complex environments, such as hydrothermal vents. Taken together, culture-dependent experiments and culture-independent genomic analysis in this study have provided comprehensive insights into the physiological features and multiple environmental adaptation strategies of *H. titanicae* in hydrothermal environments.

## Data Availability Statement

The datasets presented in this study can be found in online repositories. The names of the repository/repositories and accession number(s) can be found in the article/Supplementary Material.

## Author Contributions

MY and X-HZ designed the experiments. RD performed the genomic analysis and comparison, analyzed the data, and wrote the manuscript. RD and DG carried out the laboratory experiments. YW and LL analyzed the data. JC isolated the strain, extracted DNA, and performed genomic sequence assembly. MY, X-HZ, RD, and JL revised the manuscript. All authors contributed to the article and approved the submitted version.

## Funding

This work was supported by projects from the National Natural Science Foundation of China (41976137 and U1706208), the National Key R&D Program of China (2018YFC0310701), and the Youth Talent Support Program of the Laboratory for Marine Ecology and Environmental Science, Pilot National Laboratory for Marine Science and Technology (Qingdao; LMEES-YTSP-2018-02-07), and the Fundamental Research Funds for the Central Universities (202172002).

## Conflict of Interest

The authors declare that the research was conducted in the absence of any commercial or financial relationships that could be construed as a potential conflict of interest.

## Publisher’s Note

All claims expressed in this article are solely those of the authors and do not necessarily represent those of their affiliated organizations, or those of the publisher, the editors and the reviewers. Any product that may be evaluated in this article, or claim that may be made by its manufacturer, is not guaranteed or endorsed by the publisher.

## Supplementary Material

Supplementary Table S1 Primer sequences of different genes for RT-qPCR.

Supplementary Table S2 Summary of genomic information of 228 *Halomonas* genomes.

Supplementary Table S3 Summary of genomic information of 14 *Halomonas* genomes.

Supplementary Table S4 Genes related to metal ion metabolism in *Halomonas titanicae* SOB56 (CP059082).

Supplementary Figure S1 KEGG annotation results for 15 *Halomonas* genomes. Categories of pathways are presented at left side of heatmap by different colors. Color of each cell refers to completeness of enzymes involved in each pathway.

Supplementary Figure S2 Comparison of functional genes in four Halomonas genomes. **(A)** Total gene annotation. **(B)** Species-specific genes annotation of each strain. Designations of functional categories: **(A)** RNA processing and modification; **(B)** chromatin structure and dynamics; **(C)** energy production and conversion; **(D)** cell cycle control, cell division, and chromosome partitioning; **(E)** amino acid transport and metabolism; **(F)** nucleotide transport and metabolism; **(G)** carbohydrate transport and metabolism; **(H)** coenzyme transport and metabolism; **(I)** lipid transport and metabolism; **(J)** translation, ribosomal structure, and biogenesis; **(K)** transcription; **(L)** replication, recombination, and repair; **(M)** cell wall/membrane/envelope biogenesis; **(N)** cell motility; **(O)** posttranslational modification, protein turnover, and chaperones; **(P)** inorganic ion transport and metabolism; **(Q)** secondary metabolites biosynthesis, transport, and catabolism; **(R)** general function prediction only; **(S)** function unknown; **(T)** signal transduction mechanisms; **(U)** intracellular trafficking, secretion, and vesicular transport; **(V)** defense mechanisms; **(W)** extracellular structures.

Supplementary Figure S3 Neighbor-joining phylogenetic tree of TsdA4151, TsdB4152, and homologous proteins. Percentage bootstrap values above 50% (1,000 replicates) are shown at branch nodes.

Supplementary Figure S4 The growth course of *Halomonas titanicae* SOB56 under different concentrations of metal ions.

Supplementary Figure S5 Sodium ion tolerance of *Halomonas titanicae* SOB56.

## References

[ref1] Adams H. Teertstra W. Koster M. Tommassen J. (2002). PspE (phage-shock protein E) of *Escherichia coli* is a rhodanese. FEBS Lett. 518, 173–176. doi: 10.1016/S0014-5793(02)02695-9, PMID: 11997041

[ref2] Ahmad I. Nygren E. Khalid F. Myint S. L. Uhlin B. E. (2020). A cyclic-di-GMP signalling network regulates biofilm formation and surface associated motility of Acinetobacter baumannii 17978. Sci. Rep. 10, 1–11. doi: 10.1038/s41598-020-58522-5 32029764 PMC7005169

[ref3] Alexandre G. Greer-Phillips S. Zhulin I. B. (2004). Ecological role of energy taxis in microorganisms. FEMS Microbiol. Rev. 28, 113–126. doi: 10.1016/j.femsre.2003.10.003, PMID: 14975533

[ref4] Altschul S. F. Madden T. L. Schäffer A. A. Zhang J. Zhang Z. Miller W.. (1997). Gapped BLAST and PSI-BLAST: a new generation of protein database search programs. Nucleic Acids Res. 25, 3389–3402. doi: 10.1093/nar/25.17.3389, PMID: 9254694 PMC146917

[ref5] Andrea S. P. Christiane D. (1998). Sirohaem sulfite reductase and other proteins encoded by genes at the dsr locus of *Chromatium vinosum* are involved in the oxidation of intracellular sulfur. Microbiology 144, 1881–1894. doi: 10.1099/00221287-144-7-1881 9695921

[ref6] Apweiler R. Bairoch A. Wu C. H. Barker W. C. Boeckmann B. Ferro S.. (2004). UniProt: the universal protein knowledgebase. Nucleic Acids Res. 32, 115D–1119D. doi: 10.1093/nar/gkh131, PMID: 14681372 PMC308865

[ref7] Arahal D. R. Ventosa A. (2006). “The Family Halomonadaceae,” in The Prokaryotes: A Handbook on the Biology of Bacteria. Volume 6: Proteobacteria: Gamma Subclass, eds. Dworkin M. Falkow S. Rosenberg E. Schleifer K.-H. Stackebrandt E. (New York, NY: Springer), 811–835.

[ref8] Arai H. Kawakami T. Osamura T. Hirai T. Sakai Y. Ishii M. (2014). Enzymatic characterization and in vivo function of five terminal oxidases in *Pseudomonas aeruginosa*. J. Bacteriol. 196, 4206–4215. doi: 10.1128/JB.02176-14, PMID: 25182500 PMC4248849

[ref9] Aziz R. K. Bartels D. Best A. A. DeJongh M. Disz T. Edwards R. A.. (2008). The RAST server: rapid annotations using subsystems technology. BMC Genomics 9:75. doi: 10.1186/1471-2164-9-75, PMID: 18261238 PMC2265698

[ref10] Balsanelli E. Baura V. A. Pedrosa F. O. Souza E. M. Monteiro R. A. (2014). Exopolysaccharide biosynthesis enables mature biofilm formation on abiotic surfaces by *Herbaspirillum seropedicae*. PLoS One 9:e110392. doi: 10.1371/journal.pone.0110392, PMID: 25310013 PMC4195743

[ref11] Barco R. A. Emerson D. Sylvan J. B. Orcutt B. N. Jacobson Meyers M. E. Ramírez G. A.. (2015). New insight into microbial iron oxidation as revealed by the proteomic profile of an obligate iron-oxidizing chemolithoautotroph. Appl. Environ. Microbiol. 81, 5927–5937. doi: 10.1128/AEM.01374-15, PMID: 26092463 PMC4551237

[ref12] Beinart R. A. Gartman A. Sanders J. G. Luther G. W. Girguis P. R. (2015). The uptake and excretion of partially oxidized sulfur expands the repertoire of energy resources metabolized by hydrothermal vent symbioses. Proc. Biol. Sci. 282:20142811. doi: 10.1098/rspb.2014.2811, PMID: 25876848 PMC4426611

[ref13] Bohin J. P. (2000). Osmoregulated periplasmic glucans in Proteobacteria. FEMS Microbiol. Lett. 186, 11–19. doi: 10.1111/j.1574-6968.2000.tb09075.x 10779706

[ref14] Bouchotroch S. Quesada E. Izquierdo I. Rodríguez M. Béjar V. (2000). Bacterial exopolysaccharides produced by newly discovered bacteria belonging to the genus *Halomonas*, isolatedfrom hypersaline habitats in Morocco. J. Ind. Microbiol. Biotechnol. 24, 374–378. doi: 10.1038/sj.jim.7000002

[ref15] Brito J. A. Denkmann K. Pereira I. A. C. Archer M. Dahl C. (2015). Thiosulfate dehydrogenase (TsdA) from *Allochromatium vinosum*: Structural and functional insights into thiosulfate oxidation*. J. Biol. Chem. 290, 9222–9238. doi: 10.1074/jbc.M114.623397, PMID: 25673691 PMC4423707

[ref16] Brown N. L. Stoyanov J. V. Kidd S. P. Hobman J. L. (2003). The MerR family of transcriptional regulators. FEMS Microbiol. Rev. 27, 145–163. doi: 10.1016/s0168-6445(03)00051-2 12829265

[ref17] Capella-Gutierrez S. Silla-Martinez J. M. Gabaldon T. (2009). trimAl: a tool for automated alignment trimming in large-scale phylogenetic analyses. Bioinformatics 25, 1972–1973. doi: 10.1093/bioinformatics/btp348, PMID: 19505945 PMC2712344

[ref18] Chang H.-Y. Ahn Y. Pace L. A. Lin M. T. Lin Y.-H. Gennis R. B. (2010). The diheme cytochrome c 4 from *Vibrio cholerae* is a natural electron donor to the respiratory cbb 3 oxygen reductase. Biochemistry 49, 7494–7503. doi: 10.1021/bi1004574, PMID: 20715760 PMC2932843

[ref19] Chen X. Yin J. Ye J. Zhang H. Che X. Ma Y.. (2017). Engineering *Halomonas bluephagenesis* TD01 for non-sterile production of poly(3-hydroxybutyrate-*co*-4-hydroxybutyrate). Bioresour. Technol. 244, 534–541. doi: 10.1016/j.biortech.2017.07.149, PMID: 28803103

[ref20] Chen Y. Chen Y. Shi C. Huang Z. Zhang Y. Li S.. (2018). SOAPnuke: a MapReduce acceleration-supported software for integrated quality control and preprocessing of high-throughput sequencing data. Gigascience 7:gix120. doi: 10.1093/gigascience/gix120 PMC5788068 29220494

[ref21] Choudhury R. Srivastava S. (2001). Zinc resistance mechanisms in bacteria. Curr. Sci. 81, 768–775.

[ref22] Contreras-Moreira B. Vinuesa P. (2013). GET_HOMOLOGUES, a versatile software package for scalable and robust microbial pangenome analysis. Appl. Environ. Microbiol. 79, 7696–7701. doi: 10.1128/AEM.02411-13, PMID: 24096415 PMC3837814

[ref23] Cort J. R. Selan U. Schulte A. Grimm F. Kennedy M. A. Dahl C. (2008). *Allochromatium vinosum* DsrC: solution-state NMR structure, redox properties, and interaction with DsrEFH, a protein essential for purple sulfur bacterial sulfur oxidation. J. Mol. Biol. 382, 692–707. doi: 10.1016/j.jmb.2008.07.022, PMID: 18656485 PMC2637153

[ref24] Dahl C. (2017). “Sulfur Metabolism in Phototrophic Bacteria,” in Modern Topics in the Phototrophic Prokaryotes: Metabolism, Bioenergetics, and Omics, ed. Hallenbeck P. C. (Cham: Springer International Publishing), 27–66.

[ref25] Dahl C. Engels S. Pott-Sperling A. S. Schulte A. Sander J. Lubbe Y.. (2005). Novel genes of the dsr gene cluster and evidence for close interaction of *Dsr* proteins during sulfur oxidation in the phototrophic sulfur bacterium *Allochromatium vinosum*. J. Bacteriol. 187, 1392–1404. doi: 10.1128/JB.187.4.1392-1404.2005, PMID: 15687204 PMC545617

[ref26] Dam B. Mandal S. Ghosh W. Das Gupta S. K. Roy P. (2007). The S4-intermediate pathway for the oxidation of thiosulfate by the chemolithoautotroph *Tetrathiobacter kashmirensis* and inhibition of tetrathionate oxidation by sulfite. Res. Microbiol. 158, 330–338. doi: 10.1016/j.resmic.2006.12.013, PMID: 17509837

[ref27] Daniela G. Ranieri R. Aldo M. Isabella D. D. (2008). Nitrite and nitrate measurement by Griess reagent in human plasma: evaluation of interferences and standardization. Methods Enzymol. 440, 440, 361–380. doi: 10.1016/S0076-6879(07)00823-3 18423230

[ref28] De Maayer P. Anderson D. Cary C. Cowan D. A. (2014). Some like it cold: understanding the survival strategies of psychrophiles. EMBO Rep. 15, 508–517. doi: 10.1002/embr.201338170, PMID: 24671034 PMC4210084

[ref29] Denkmann K. Grein F. Zigann R. Siemen A. Bergmann J. van Helmont S.. (2012). Thiosulfate dehydrogenase: a widespread unusual acidophilic *c*-type cytochrome. Environ. Microbiol. 14, 2673–2688. doi: 10.1111/j.1462-2920.2012.02820.x, PMID: 22779704

[ref30] Dick G. J. (2019). The microbiomes of deep-sea hydrothermal vents: distributed globally, shaped locally. Microbiology 17, 271–283. doi: 10.1038/s41579-019-0160-2, PMID: 30867583

[ref31] Doroshchuk N. A. Gelfand M. S. Rodionov D. A. (2006). Regulation of nitrogen metabolism in gram-positive bacteria. Mol. Biol. 40, 829–836. doi: 10.1134/s0026893306050190 17086994

[ref32] Feller G. (2013). Psychrophilic enzymes: from folding to function and biotechnology. Scientifica (Cairo) 2013:512840. doi: 10.1155/2013/512840, PMID: 24278781 PMC3820357

[ref33] Felske A. D. Fehr W. Pauling B. V. Canstein V. H. Wagner-Döbler I. (2003). Functional profiling of mercuric reductase (*mer* A) genes in biofilm communities of a technical scale biocatalyzer. BMC Microbiol. 3, 22–11. doi: 10.1186/1471-2180-3-22 14577839 PMC270059

[ref34] Finn R. D. Bateman A. Clements J. Coggill P. Eberhardt R. Y. Eddy S. R.. (2014). Pfam: the protein families database. Nucleic Acids Res. 42, D222–D230. doi: 10.1093/nar/gkt1223, PMID: 24288371 PMC3965110

[ref35] Florindo C. Ferreira R. Borges V. Spellerberg B. Gomes J. P. Borrego M. J. (2012). Selection of reference genes for real-time expression studies in *Streptococcus agalactiae*. J. Microbiol. Methods 90, 220–227. doi: 10.1016/j.mimet.2012.05.011, PMID: 22634000

[ref36] Fortunato C. S. Larson B. Butterfield D. A. Huber J. A. (2018). Spatially distinct, temporally stable microbial populations mediate biogeochemical cycling at and below the seafloor in hydrothermal vent fluids. Environ. Microbiol. 20, 769–784. doi: 10.1111/1462-2920.14011, PMID: 29205750

[ref37] García-Estepa R. Argandona M. Reina-Bueno M. Capote N. Iglesias-Guerra F. Nieto J. J.. (2006). The *ectD* gene, which is involved in the synthesis of the compatible solute hydroxyectoine, is essential for thermoprotection of the halophilic bacterium *Chromohalobacter salexigens*. J. Bacteriol. 188, 3774–3784. doi: 10.1128/JB.00136-06, PMID: 16707670 PMC1482885

[ref38] Gartman A. Yücel M. Madison A. S. Chu D. W. Ma S. Janzen C. P.. (2011). Sulfide oxidation across diffuse flow zones of hydrothermal vents. Aquat. Geochem. 17, 583–601. doi: 10.1007/s10498-011-9136-1

[ref39] Gasteiger E. Hoogland C. Gattiker A. Duvaud S.e. Wilkins M. R. Appel R. D.. (2005). “Protein Identification and Analysis Tools on the ExPASy Server,” in The Proteomics Protocols Handbook, ed. Walker J. M. (Totowa, NJ: Humana Press), 571–607.

[ref40] Ghosh W. Dam B. (2009). Biochemistry and molecular biology of lithotrophic sulfur oxidation by taxonomically and ecologically diverse bacteria and archaea. FEMS Microbiol. Rev. 33, 999–1043. doi: 10.1111/j.1574-6976.2009.00187.x, PMID: 19645821

[ref41] Ghosh W. Mazumdar A. Peketi A. Fernandes S. Roy C. (2020). Cryptic roles of tetrathionate in the sulfur cycle of marine sediments: microbial drivers and indicators. Biogeosciences 17, 4611–4631. doi: 10.5194/bg-17-4611-2020

[ref42] Grammann K. Volke A. Kunte H. J. (2002). New type of osmoregulated solute transporter identified in halophilic members of the bacteria domain: TRAP transporter TeaABC mediates uptake of ectoine and hydroxyectoine in *Halomonas elongata* DSM 2581^T^. J. Bacteriol. 184, 3078–3085. doi: 10.1128/JB.184.11.3078-3085.2002, PMID: 12003950 PMC135061

[ref43] Hensen D. Sperling D. Truper H. G. Brune D. C. Dahl C. (2006). Thiosulphate oxidation in the phototrophic sulphur bacterium *Allochromatium vinosum*. Mol. Microbiol. 62, 794–810. doi: 10.1111/j.1365-2958.2006.05408.x, PMID: 16995898

[ref44] Hickman J. W. Tifrea D. F. Harwood C. S. (2005). A chemosensory system that regulates biofilm formation through modulation of cyclic diguanylate levels. Proc. Natl. Acad. Sci. 102, 14422–14427. doi: 10.1073/pnas.0507170102, PMID: 16186483 PMC1234902

[ref45] Huo Y. Y. Li Z. Y. Cheng H. Wang C. S. Xu X. W. (2014). High quality draft genome sequence of the heavy metal resistant bacterium *Halomonas zincidurans* type strain B6^T^. Stand Genomic Sci. 9, 1–9. doi: 10.1186/1944-3277-9-30 25945155 PMC4286145

[ref46] Ikeuchi Y. Shigi N. Kato J. Nishimura A. Suzuki T. (2006). Mechanistic insights into sulfur relay by multiple sulfur mediators involved in thiouridine biosynthesis at tRNA wobble positions. Mol. Cell 21, 97–108. doi: 10.1016/j.molcel.2005.11.001, PMID: 16387657

[ref47] Jenner L. P. Kurth J. M. van Helmont S. Sokol K. P. Reisner E. Dahl C.. (2019). Heme ligation and redox chemistry in two bacterial thiosulfate dehydrogenase (TsdA) enzymes. J. Biol. Chem. 294, 18002–18014. doi: 10.1074/jbc.RA119.010084, PMID: 31467084 PMC6879331

[ref48] Kanehisa M. Goto S. Kawashima S. Okuno Y. Hattori M. (2004). The KEGG resource for deciphering the genome. Nucleic Acids Res. 32, 277D–280D. doi: 10.1093/nar/gkh063, PMID: 14681412 PMC308797

[ref49] Kaye J. Z. Baross J. A. (2000). High incidence of halotolerant bacteria in Pacific hydrothermal-vent and pelagic environments. FEMS Microbiol. Ecol. 32, 249–260. doi: 10.1111/j.1574-6941.2000.tb00718.x, PMID: 10858584

[ref50] Kaye J. Z. Marquez M. C. Ventosa A. Baross J. A. (2004). *Halomonas neptunia* sp. nov., *Halomonas sulfidaeris* sp. nov., *Halomonas axialensis* sp. nov. and *Halomonas hydrothermalis* sp. nov.: halophilic bacteria isolated from deep-sea hydrothermal-vent environments. Int. J. Syst. Evol. Microbiol. 54, 499–511. doi: 10.1099/ijs.0.02799-0, PMID: 15023967

[ref51] Kazutaka K. Kazuharu M. Kei-ichi K. Takashi M. (2002). MAFFT: a novel method for rapid multiple sequence alignment based on fast Fourier transform. Nucleic Acids Res. 30, 3059–3066. doi: 10.1093/nar/gkf436 12136088 PMC135756

[ref52] Kentaro N. Ken T. (2014). Theoretical constraints of physical and chemical properties of hydrothermal fluids on variations in chemolithotrophic microbial communities in seafloor hydrothermal systems. Prog. Earth Planet. Sci. 1, 24. doi: 10.1186/2197-4284-1-5

[ref53] Koren S. Walenz B. P. Berlin K. Miller J. R. Bergman N. H. Phillippy A. M. (2017). Canu: scalable and accurate long-read assembly via adaptive *k*-mer weighting and repeat separation. Genome Res. 27, 722–736. doi: 10.1101/gr.215087.116, PMID: 28298431 PMC5411767

[ref54] Kredich N. M. (1992). The molecular basis for positive regulation of cys promoters in *Salmonella typhimurium* and *Escherichia coli*. Mol. Microbiol. 6, 2747–2753. doi: 10.1111/j.1365-2958.1992.tb01453.x, PMID: 1435253

[ref55] Kristensen D. M. Kannan L. Coleman M. K. Wolf Y. I. Sorokin A. Koonin E. V.. (2010). A low-polynomial algorithm for assembling clusters of orthologous groups from intergenomic symmetric best matches. Bioinformatics 26, 1481–1487. doi: 10.1093/bioinformatics/btq229, PMID: 20439257 PMC2881409

[ref56] Krogh A. Larsson B. Heijne G. V. Sonnhammer E. L. (2001). Predicting transmembrane protein topology with a hidden Markov model: application to complete genomes. J. Mol. Biol. 305, 567–580. doi: 10.1006/jmbi.2000.4315, PMID: 11152613

[ref57] Kuhlmann A. U. Bremer E. (2002). Osmotically regulated synthesis of the compatible solute ectoine in *Bacillus pasteurii* and related *Bacillus* spp. Appl. Environ. Microbiol. 68, 772–783. doi: 10.1128/AEM.68.2.772-783.2002, PMID: 11823218 PMC126723

[ref58] Kumar S. Stecher G. Tamura K. (2016). MEGA7: molecular evolutionary genetics analysis version 7.0 for bigger datasets. Mol. Biol. Evol. 33, 1870–1874. doi: 10.1093/molbev/msw054, PMID: 27004904 PMC8210823

[ref59] Kurth J. M. Brito J. A. Reuter J. Flegler A. Koch T. Franke T.. (2016). Electron accepting units of the diheme cytochrome c TsdA, a bifunctional thiosulfate dehydrogenase/tetrathionate reductase. J. Biol. Chem. 291, 24804–24818. doi: 10.1074/jbc.M116.753863, PMID: 27694441 PMC5122753

[ref60] Kushwaha B. Jadhav I. Verma H. N. Geethadevi A. Parashar D. Jadhav K. (2019). Betaine accumulation suppresses the *de-novo* synthesis of ectoine at a low osmotic concentration in *Halomonas* sp SBS 10, a bacterium with broad salinity tolerance. Mol. Biol. Rep. 46, 4779–4786. doi: 10.1007/s11033-019-04924-2, PMID: 31230183

[ref61] Larkin M. A. Blackshields G. Brown N. P. Chenna R. McGettigan P. A. McWilliam H.. (2007). Clustal W and Clustal X version 2.0. Bioinformatics 23, 2947–2948. doi: 10.1093/bioinformatics/btm404, PMID: 17846036

[ref62] Leigh J. A. Dodsworth J. A. (2007). Nitrogen regulation in bacteria and archaea. Annu. Rev. Microbiol. 61, 349–377. doi: 10.1146/annurev.micro.61.080706.093409 17506680

[ref63] Li B. Liu J. Zhou S. Fu L. Yao P. Chen L.. (2019). Vertical variation in *Vibrio* community composition in Sansha Yongle blue hole and its ability to degrade macromolecules. Mar. Life Sci. Technol. 2, 60–72. doi: 10.1007/s42995-019-00003-4

[ref64] Li L. Stoeckert C. J. Roos D. S. (2003). OrthoMCL: identification of ortholog groups for eukaryotic genomes. Genome Res. 13, 2178–2189. doi: 10.1101/gr.1224503, PMID: 12952885 PMC403725

[ref65] Li Y. Tang K. Zhang L. Zhao Z. Xie X. Chen C. A.. (2018). Coupled carbon, sulfur, and nitrogen cycles mediated by microorganisms in the water column of a shallow-water hydrothermal ecosystem. Front. Microbiol. 9:2718. doi: 10.3389/fmicb.2018.02718, PMID: 30555427 PMC6282030

[ref66] Mandal S. Rameez M. J. Chatterjee S. Sarkar J. Pyne P. Bhattacharya S.. (2020). Molecular mechanism of sulfur chemolithotrophy in the betaproteobacterium *Pusillimonas ginsengisoli* SBSA. Microbiology (Reading) 166, 386–397. doi: 10.1099/mic.0.000890, PMID: 31999239

[ref67] Mata J. A. Martinez-Canovas J. Quesada E. Bejar V. (2002). A detailed phenotypic characterisation of the type strains of *Halomonas* species. Syst. Appl. Microbiol. 25, 360–375. doi: 10.1078/0723-2020-00122, PMID: 12421074

[ref68] Mata J. A. Béjar V. Llamas I. Arias S. Bressollier P. Tallon R.. (2006). Exopolysaccharides produced by the recently described halophilic bacteria *Halomonas ventosae* and *Halomonas anticariensis*. Res. Microbiol. 157, 827–835. doi: 10.1016/j.resmic.2006.06.004, PMID: 17005380

[ref69] Minh B. Q. Schmidt H. A. Chernomor O. Schrempf D. Woodhams M. D. von Haeseler A.. (2020). IQ-TREE 2: new models and efficient methods for phylogenetic inference in the genomic era. Mol. Biol. Evol. 37, 1530–1534. doi: 10.1093/molbev/msaa015, PMID: 32011700 PMC7182206

[ref70] Miyazaki J. Kawagucci S. Makabe A. Takahashi A. Kitada K. Torimoto J.. (2017). Deepest and hottest hydrothermal activity in the Okinawa Trough: the Yokosuka site at Yaeyama knoll. R. Soc. Open Sci. 4:171570. doi: 10.1098/rsos.171570, PMID: 29308272 PMC5750039

[ref71] Mullaugh K. M. Luther G. W. Ma S. Moore T. S. Yücel M. Becker E. L.. (2008). Voltammetric (Micro)electrodes for the In situ study of Fe^2+^ oxidation kinetics in Hot Springs and S_2_O_3_^2−^ production at hydrothermal vents. Electroanalysis 20, 280–290. doi: 10.1002/elan.200704056

[ref72] Muro-Pastor M. I. Reyes J. C. Florencio F. J. (2005). Ammonium assimilation in cyanobacteria. Photosynth. Res. 83, 135–150. doi: 10.1007/s11120-004-2082-7 16143848

[ref73] Nakagawa S. Takai K. (2008). Deep-sea vent chemoautotrophs: diversity, biochemistry and ecological significance. FEMS Microbiol. Ecol. 65, 1–14. doi: 10.1111/j.1574-6941.2008.00502.x, PMID: 18503548

[ref74] Nies D. H. (1999). Microbial heavy-metal resistance. Appl. Microbiol. Biotechnol. 51, 730–750. doi: 10.1007/s002530051457 10422221

[ref75] Patwardhan S. Foustoukos D. I. Giovannelli D. Yucel M. Vetriani C. (2018). Ecological succession of sulfur-oxidizing epsilon- and gammaproteobacteria during colonization of a shallow-water gas vent. Front. Microbiol. 9:2970. doi: 10.3389/fmicb.2018.02970, PMID: 30574130 PMC6291522

[ref76] Petersen T. N. Brunak S. Heijne G.v. Nielsen H. (2011). SignalP 4.0: discriminating signal peptides from transmembrane regions. Nat. Methods 8, 785–786. doi: 10.1038/nmeth.1701, PMID: 21959131

[ref77] Poli A. Nicolaus B. Denizci A. A. Yavuzturk B. Kazan D. (2013). *Halomonas smyrnensis* sp. nov., a moderately halophilic, exopolysaccharide-producing bacterium. Int. J. Syst. Evol. Microbiol. 63, 10–18. doi: 10.1099/ijs.0.037036-0, PMID: 22328606

[ref78] Pruitt K. D. Tatusova T. Maglott D. R. (2007). NCBI reference sequences (RefSeq): a curated non-redundant sequence database of genomes, transcripts and proteins. Nucleic Acids Res. 35, D61–D65. doi: 10.1093/nar/gkl842, PMID: 17130148 PMC1716718

[ref79] Pyne P. Alam M. Rameez M. J. Mandal S. Sar A. Mondal N.. (2018). Homologs from sulfur oxidation (sox) and methanol dehydrogenation (Xox) enzyme systems collaborate to give rise to a novel pathway of chemolithotrophic tetrathionate oxidation. Mol. Microbiol. 109, 169–191. doi: 10.1111/mmi.13972, PMID: 29669166

[ref80] Rameez M. J. Pyne P. Mandal S. Chatterjee S. Alam M. Bhattacharya S.. (2020). Two pathways for thiosulfate oxidation in the alphaproteobacterial chemolithotroph *Paracoccus thiocyanatus* SST. Microbiol. Res. 230:126345. doi: 10.1016/j.micres.2019.126345, PMID: 31585234

[ref81] Reitzer L. (2003). Nitrogen assimilation and global regulation in *Escherichia coli*. Annu. Rev. Microbiol. 57, 155–176. doi: 10.1146/annurev.micro.57.030502.090820 12730324

[ref82] Richter M. Rossello-Mora R. Oliver G. F. Peplies J. (2016). JSpeciesWS: a web server for prokaryotic species circumscription based on pairwise genome comparison. Bioinformatics 32, 929–931. doi: 10.1093/bioinformatics/btv681, PMID: 26576653 PMC5939971

[ref83] Robert X. Gouet P. (2014). Deciphering key features in protein structures with the new ENDscript server. Nucleic Acids Res. 42, W320–W324. doi: 10.1093/nar/gku316, PMID: 24753421 PMC4086106

[ref84] Roman L. T. Michael Y. G. Darren A. N. Eugene V. K. (2000). The COG database: a tool for genome-scale analysis of protein functions and evolution. Nucleic Acids Res. 28, 33–36. doi: 10.1093/nar/28.1.33 10592175 PMC102395

[ref85] Ruby E. Wirsen C. Jannasch H. (1981). Chemolithotrophic sulfur-oxidizing bacteria from the galapagos rift hydrothermal vents. Appl. Environ. Microbiol. 42, 317–324. doi: 10.1128/aem.42.2.317-324.1981, PMID: 16345831 PMC244008

[ref86] Sánchez-Porro C. Kaur B. Mann H. Ventosa A. (2010). *Halomonas titanicae* sp. nov., a halophilic bacterium isolated from the RMS *titanic*. Int. J. Syst. Evol. Microbiol. 60, 2768–2774. doi: 10.1099/ijs.0.020628-0, PMID: 20061494

[ref87] Scott K. M. Williams J. Porter C. M. B. Russel S. Harmer T. L. Paul J. H.. (2018). Genomes of ubiquitous marine and hypersaline *Hydrogenovibrio*, *Thiomicrorhabdus* and *Thiomicrospira* spp. encode a diversity of mechanisms to sustain chemolithoautotrophy in heterogeneous environments. Environ. Microbiol. 20, 2686–2708. doi: 10.1111/1462-2920.14090, PMID: 29521452

[ref88] Shao M. F. Zhang T. Fang H. H. (2010). Sulfur-driven autotrophic denitrification: diversity, biochemistry, and engineering applications. Appl. Microbiol. Biotechnol. 88, 1027–1042. doi: 10.1007/s00253-010-2847-1, PMID: 20809074

[ref89] Simon-Colin C. Raguenes G. Cozien J. Guezennec J. G. (2008). *Halomonas profundus* sp. nov., a new PHA-producing bacterium isolated from a deep-sea hydrothermal vent shrimp. J. Appl. Microbiol. 104, 1425–1432. doi: 10.1111/j.1365-2672.2007.03667.x, PMID: 18179545

[ref90] Sinha R. K. Krishnan K. P. Thomas F. A. Binish M. B. Mohan M. Kurian P. J. (2019). Polyphasic approach revealed complex bacterial community structure and function in deep sea sediment of ultra-slow spreading Southwest Indian Ridge. Ecol. Indic. 96, 40–51. doi: 10.1016/j.ecolind.2018.08.063

[ref91] Sleator R. D. Hill C. (2002). Bacterial osmoadaptation: the role of osmolytes in bacterial stress and virulence. FEMS Microbiol. Rev. 26, 49–71. doi: 10.1111/j.1574-6976.2002.tb00598.x, PMID: 12007642

[ref92] Sone Y. Pan-Hou H. Nakamura R. Sakabe K. Kiyono M. (2010). Roles played by MerE and MerT in the transport of inorganic and organic mercury compounds in gram-negative bacteria. J. Health Sci. 56, 123–127. doi: 10.1248/jhs.56.123

[ref93] Sorokin D. Y. (2003). Oxidation of inorganic sulfur compounds by obligately organotrophic bacteria. Microbiology 72, 641–653. doi: 10.1023/B:MICI.0000008363.24128.e5 14768537

[ref94] Sorokin D. Y. Tourova T. P. Muyzer G. (2005). Oxidation of thiosulfate to tetrathionate by an haloarchaeon isolated from hypersaline habitat. Extremophiles 9, 501–504. doi: 10.1007/s00792-005-0465-0, PMID: 16041477

[ref95] Sperandio V. Torres A. G. Kaper J. B. (2002). Quorum sensing *Escherichia coli* regulators B and C (QseBC): a novel two-component regulatory system involved in the regulation of flagella and motility by quorum sensing in *E. coli*. Mol. Microbiol. 43, 809–821. doi: 10.1046/j.1365-2958.2002.02803.x, PMID: 11929534

[ref96] Stamatakis A. (2006). RAxML-VI-HPC: maximum likelihood-based phylogenetic analyses with thousands of taxa and mixed models. Bioinformatics 22, 2688–2690. doi: 10.1093/bioinformatics/btl446, PMID: 16928733

[ref97] Sievert S. M. Hügler M. Taylor C. D. Wirsen C. O. (2008). “Sulfur Oxidation at Deep-Sea Hydrothermal Vents,” in Microbial Sulfur Metabolism, eds. Dahl C. Friedrich C. G. (Berlin, Heidelberg: Springer), 238–258.

[ref98] Suzuki A. Knaff D. B. (2005). Glutamate synthase: structural, mechanistic and regulatory properties, and role in the amino acid metabolism. Photosynth. Res. 83, 191–217. doi: 10.1007/s11120-004-3478-0, PMID: 16143852

[ref99] Tang K. Liu K. Jiao N. Zhang Y. Chen C. T. (2013). Functional metagenomic investigations of microbial communities in a shallow-sea hydrothermal system. PLoS One 8:e72958. doi: 10.1371/journal.pone.0072958, PMID: 23940820 PMC3735525

[ref100] Taylor B. L. Zhulin I. B. (1999). PAS domains: internal sensors of oxygen, redox potential, and light. Microbiol. Mol. Biol. Rev. 63, 479–506. doi: 10.1128/MMBR.63.2.479-506.1999, PMID: 10357859 PMC98974

[ref101] Tsai C. M. Frasch C. E. (1982). A sensitive silver stain for detecting lipopolysaccharides in polyacrylamide gels. Anal. Biochem. 119, 115–119. doi: 10.1016/0003-2697(82)90673-X, PMID: 6176137

[ref102] Vetriani C. Chew Y. S. Miller S. M. Yagi J. Coombs J. Lutz R. A.. (2005). Mercury adaptation among bacteria from a deep-sea hydrothermal vent. Appl. Environ. Microbiol. 71, 220–226. doi: 10.1128/AEM.71.1.220-226.2005, PMID: 15640191 PMC544242

[ref103] Walker B. J. Abeel T. Shea T. Priest M. Abouelliel A. Sakthikumar S.. (2014). Pilon: an integrated tool for comprehensive microbial variant detection and genome assembly improvement. PLoS One 9:e112963. doi: 10.1371/journal.pone.0112963, PMID: 25409509 PMC4237348

[ref01] Wang Y. Li H. Cui X. Zhang X.-H. (2017). A novel stress response mechanism, triggered by indole, involved in quorum quenching enzyme MomL and iron-sulfur cluster in *Muricauda olearia* Th120. Sci. Rep. 7, 1–10. doi: 10.1038/s41598-017-04606-8, PMID: 28652609 PMC5484670

[ref106] Wang R. Lin J. Q. Liu X. M. Pang X. Zhang C. J. Yang C. L.. (2018a). Sulfur oxidation in the acidophilic autotrophic Acidithiobacillus spp. Front. Microbiol. 9:3290. doi: 10.3389/fmicb.2018.03290, PMID: 30687275 PMC6335251

[ref104] Wang L. Shao Z. (2021). Aerobic denitrification and heterotrophic sulfur oxidation in the genus Halomonas revealed by six novel species characterizations and genome-based analysis. Front. Microbiol. 12:652766. doi: 10.3389/fmicb.2021.652766 33815342 PMC8014003

[ref105] Wang L. Yu M. Liu Y. Liu J. Wu Y. Li L.. (2018b). Comparative analyses of the bacterial community of hydrothermal deposits and seafloor sediments across Okinawa Trough. J. Mar. Syst. 180, 162–172. doi: 10.1016/j.jmarsys.2016.11.012

[ref107] Wargo M. J. Szwergold B. S. Hogan D. A. (2008). Identification of two gene clusters and a transcriptional regulator required for *Pseudomonas aeruginosa* glycine betaine catabolism. J. Bacteriol. 190, 2690–2699. doi: 10.1128/JB.01393-07, PMID: 17951379 PMC2293255

[ref108] Williams T. J. Lauro F. M. Ertan H. Burg D. W. Poljak A. Raftery M. J.. (2011). Defining the response of a microorganism to temperatures that span its complete growth temperature range (−2°C to 28°C) using multiplex quantitative proteomics. Environ. Microbiol. 13, 2186–2203. doi: 10.1111/j.1462-2920.2011.02467.x, PMID: 21443741

[ref109] Xu L. Xu X. W. Meng F. X. Huo Y. Y. Oren A. Yang J. Y.. (2013). *Halomonas zincidurans* sp. nov., a heavy-metal-tolerant bacterium isolated from the deep-sea environment. Int. J. Syst. Evol. Microbiol. 63, 4230–4236. doi: 10.1099/ijs.0.051656-0, PMID: 23811134

[ref110] Yamamoto M. Takai K. (2011). Sulfur metabolisms in epsilon- and gamma-proteobacteria in deep-sea hydrothermal fields. Front. Microbiol. 2:192. doi: 10.3389/fmicb.2011.00192, PMID: 21960986 PMC3176464

[ref111] Yang Z. Xiao X. Zhang Y. (2019). Microbial diversity of sediments from an inactive hydrothermal vent field, Southwest Indian Ridge. Mar. Life Sci. Technol. 2, 73–86. doi: 10.1007/s42995-019-00007-0

[ref112] Yin Q. Fu B. Li B. Shi X. Inagaki F. Zhang X. H. (2013). Spatial variations in microbial community composition in surface seawater from the ultra-oligotrophic center to rim of the South Pacific Gyre. PLoS One 8:e55148. doi: 10.1371/journal.pone.0055148, PMID: 23405118 PMC3566182

[ref113] Zhang H. Dreisinger D. B. (2002). The kinetics for the decomposition of tetrathionate in alkaline solutions. Hydrometallurgy 66, 59–65. doi: 10.1016/S0304-386X(02)00078-6

[ref114] Zhang J. Liu R. Xi S. Cai R. Zhang X. Sun C. (2020). A novel bacterial thiosulfate oxidation pathway provides a new clue about the formation of zero-valent sulfur in deep sea. ISME J. 14, 2261–2274. doi: 10.1038/s41396-020-0684-5, PMID: 32457501 PMC7608252

[ref115] Zhao B. Wang H. Mao X. Li R. Zhang Y. J. Tang S.. (2012). *Halomonas xianhensis* sp. nov., a moderately halophilic bacterium isolated from a saline soil contaminated with crude oil. Int. J. Syst. Evol. Microbiol. 62, 173–178. doi: 10.1099/ijs.0.025627-0, PMID: 21378136

