# Supplementary material for: Heterotrophic Sulfur Oxidation of Halomonas titanicae SOB56 and Its Habitat Adaptation to the Hydrothermal Environment
Source: Front Microbiol. 2022 Jun 14;13:888833. doi: 10.3389/fmicb.2022.888833 (PMC9237845; doi:10.3389/fmicb.2022.888833)
Supplement: Supplementary Table S1 — Primer sequences of different genes for RT-qPCR. [file Data_Sheet_1.pdf]

Supplementary Materials

SUPPLEMENTARY TABLES

Supplementary Table 1. Primer sequences of different genes for RT-qPCR

Name	Primer sequences
Q4151-F	GTATCGCAGGTTGCTTTGCTTATT
Q4151-R	GAACAGTTTTCCTGGTAAGCCG
Q4152-F	ATGATGACGTTTGCCAATATGCT
Q4152-R	TGATGCGTGTCTCGTTTTTCCA
RecA-F	TGAAGTTCTATGCCAGCGTGCG
RecA-R	TGGCTTTACCTTGACCGATTTTCTT

Supplementary Table 4. Genes related to metal ion metabolism in *H. titanicae* SOB56 (CP059082)

Metal Element	Encoded gene product
Cu	Copper homeostasis protein CutE
	Copper resistance protein CopC
	Copper resistance protein CopD
	Copper resistance protein CopB
	Multicopper oxidase
Fe	Iron (III) dicitrate transport system permease protein FecD
	ABC transporter (iron), permease component
	Iron-chelator utilization protein
	Iron-regulated protein A precursor
	Iron-uptake factor PiuB
	Ferric iron ABC transporter, ATP-binding protein
	Ferric iron ABC transporter, permease protein
	Ferroxidase
	ABC-type Fe^{3+}-siderophore transport system, periplasmic iron-binding component
Mn	Manganese ABC transporter, periplasmic-binding protein SitA
	Manganese ABC transporter, ATP-binding protein SitB
	Manganese ABC transporter, inner membrane permease protein SitC
	Manganese ABC transporter, inner membrane permease protein SitD
	Manganese superoxide dismutase (EC 1.15.1.1)
As	Arsenical-resistance protein ACR3
	Arsenical resistance operon repressor
	Arsenic resistance protein ArsH
	Arsenic efflux pump protein
Ni	Nickel responsive regulator NikR
Zn	Lead, cadmium, zinc and mercury transporting ATPase
	Zinc uptake regulation protein ZUR
	Zinc ABC transporter, periplasmic-binding protein ZnuA
	Zinc ABC transporter, inner membrane permease protein ZnuB
	Zinc ABC transporter, ATP-binding protein ZnuC
	Exported zinc metalloprotease YfgC precursor
	Membrane-associated zinc metalloprotease
Mg/Co	Magnesium and cobalt transport protein CorA
	Magnesium and cobalt efflux protein CorC
	Cobalt ABC transporter, ATP-binding component CbtL
Co-Zn-Cd	Cobalt-zinc-cadmium resistance protein CzcA; Cation efflux system protein CusA
	Cobalt-zinc-cadmium resistance protein CzcD

Pb/Cd/Zn/Hg	Lead, cadmium, zinc and mercury transporting ATPase
Hg	Mercuric transport protein, MerE
	Mercuric resistance operon coregulator
	Mercuric ion reductase (EC 1.16.1.1)
	Mercuric transport protein, MerC
	Periplasmic mercury (+2) binding protein
	Mercuric transport protein, MerT
	Mercuric resistance operon regulatory protein
Mo	Molybdenum ABC transporter, periplasmic molybdenum-binding protein ModA (TC 3.A.1.8.1)
	Molybdenum transport system permease protein ModB (TC 3.A.1.8.1)
	Molybdenum transport ATP-binding protein ModC (TC 3.A.1.8.1)
	Putative molybdenum transport ATP-binding protein modF
Others	Metal transporter, ZIP family
	Heavy metal sensor histidine kinase

SUPPLEMENTARY FIGURES

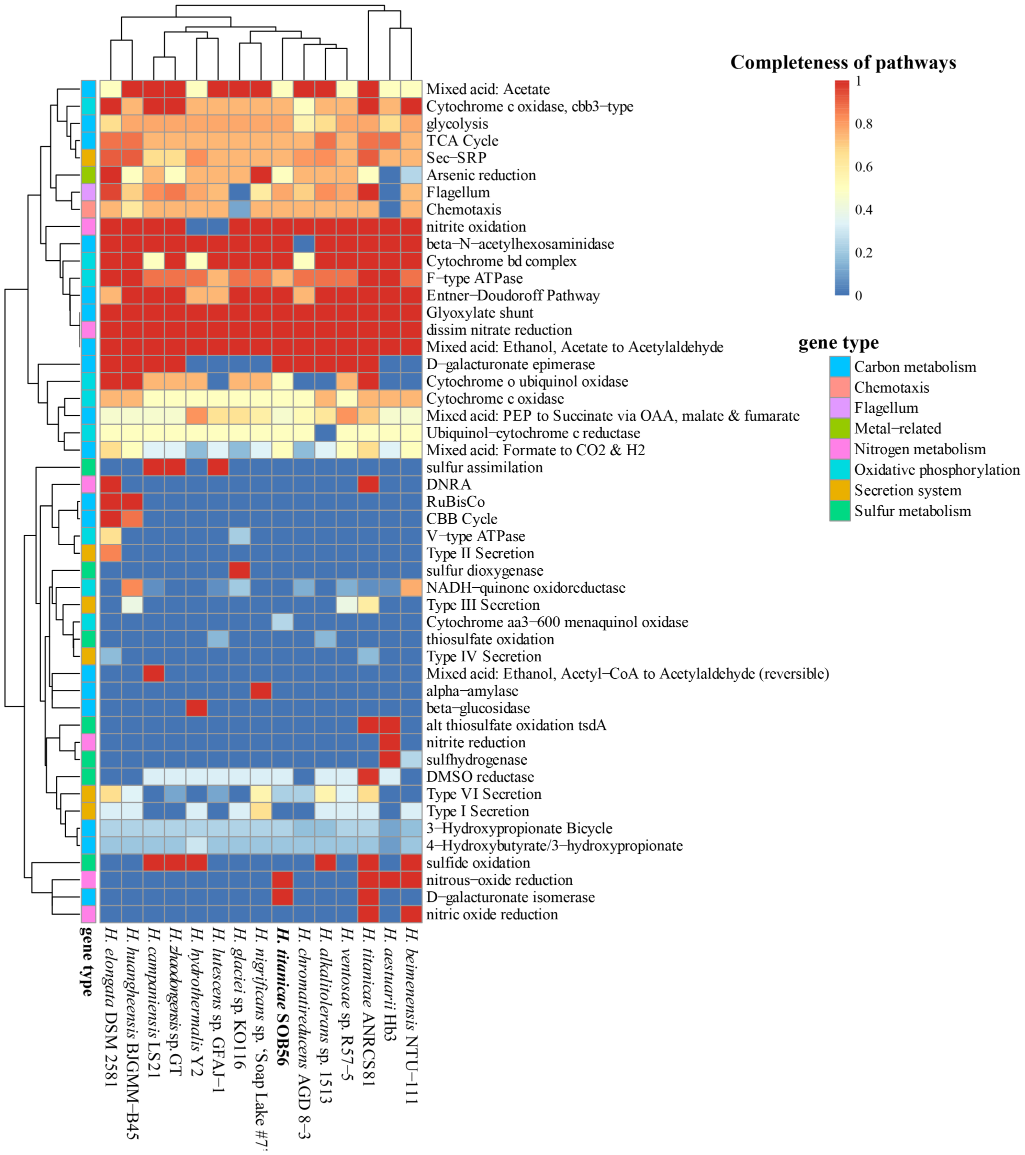

Supplementary Figure 1. KEGG annotation results for fifteen *Halomonas* genomes. Categories of pathways are presented at left side of heatmap by different colors. Color of each cell refers to completeness of enzymes involved in each pathway.

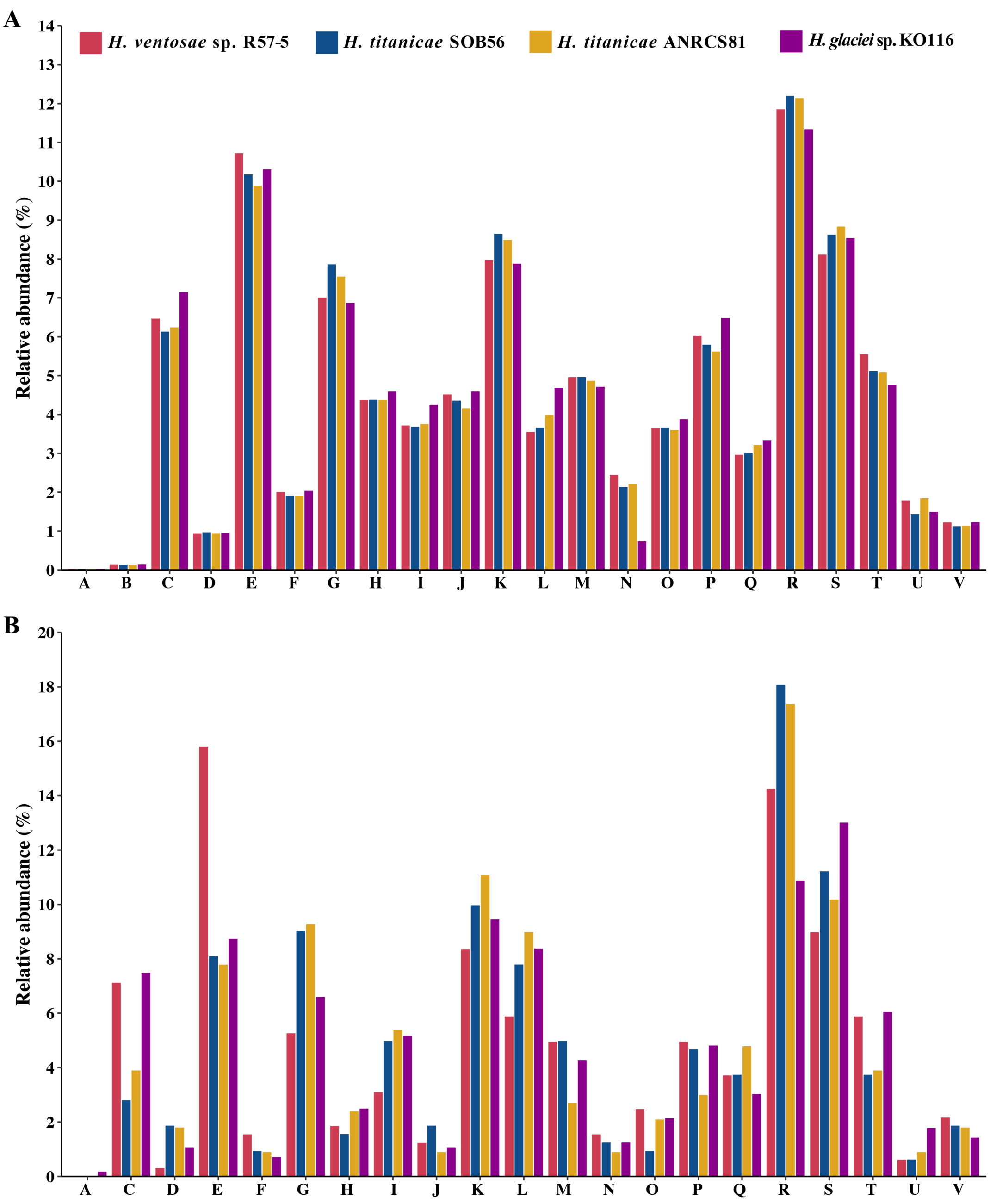

Supplementary Figure 2. Comparison of functional genes in four *Halomonas* genomes. **A** Total gene annotation. **B** Species-specific genes annotation of each strain. Designations of functional categories: A, RNA processing and modification; B, chromatin structure and dynamics; C, energy production and conversion; D, cell cycle control, cell division, chromosome partitioning; E, amino acid transport and metabolism; F, nucleotide transport and metabolism; G, carbohydrate transport and metabolism; H, coenzyme transport and metabolism; I, lipid transport and metabolism; J, translation, ribosomal structure and biogenesis; K, transcription; L, replication, recombination and repair; M, cell wall/membrane/envelope biogenesis; N, cell motility; O, posttranslational modification, protein turnover, chaperones; P, inorganic ion transport and metabolism; Q, secondary metabolites biosynthesis, transport and catabolism; R, general function prediction only; S, function unknown; T, signal

transduction mechanisms; U, intracellular trafficking, secretion, and vesicular transport; V, defense mechanisms; W, extracellular structures.

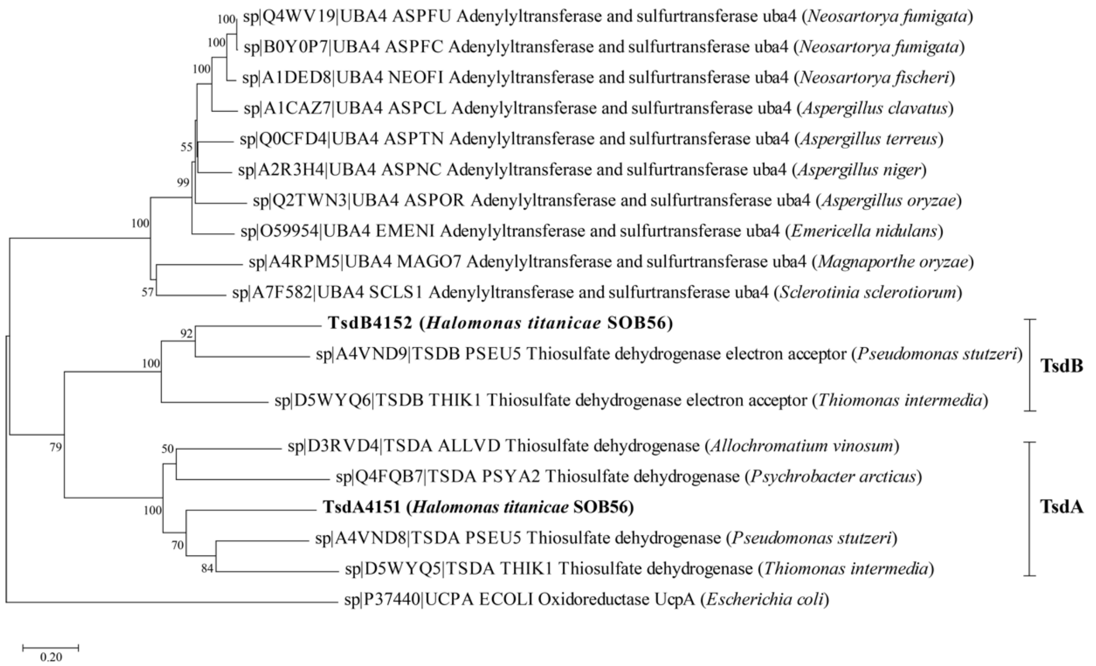

Supplementary Figure 3. Neighbor-joining phylogenetic tree of TsdA4151, TsdB4152 and homologous proteins. Percentage bootstrap values above 50% (1000 replicates) are shown at branch nodes.

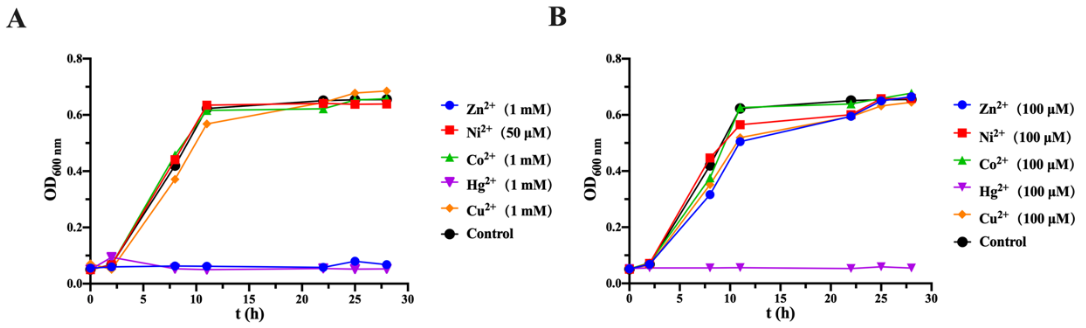

Supplementary Figure 4. The growth course of *H. titanicae* SOB56 under different concentrations of metal ions.

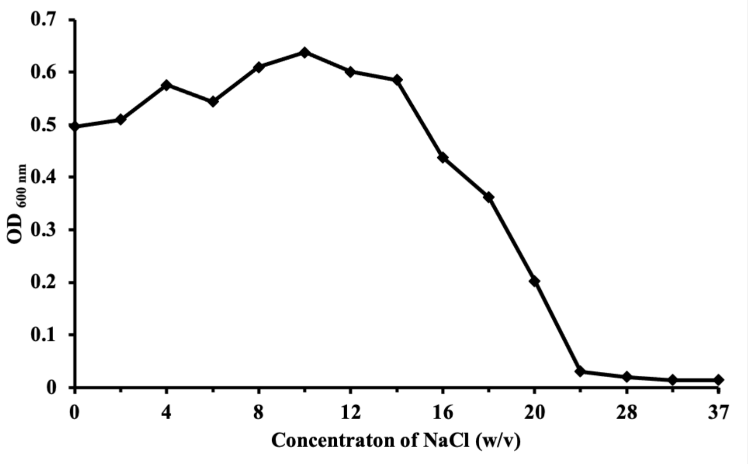

Supplementary Figure 5. Sodium ion tolerance of *H. titanicae* SOB56.
